# Genetic causal relationship between gut microbiome and psoriatic arthritis: a bidirectional two-sample Mendelian randomization study

**DOI:** 10.3389/fmicb.2023.1265786

**Published:** 2023-10-31

**Authors:** Xinyu Qian, Zhida Fu, Chaoyue Diao, Wenbo Zhang, Weiyu Tao, Jiaqi Hu, Shuqing Zhang, Dongbao Zhao

**Affiliations:** ^1^Department of Rheumatology and Immunology, Changhai Hospital, Naval Medical University, Shanghai, China; ^2^Department of Reproductive Medicine, Changhai Hospital, Naval Medical University, Shanghai, China

**Keywords:** psoriatic arthritis, causal relationship, genetics, gut microbiome, Mendelian randomization

## Abstract

**Background:**

Several observational studies have suggested a potential relationship between gut microbiome and psoriatic arthritis (PsA). However, the causality of this relationship still remains unclear. We aim to explore if the specific gut microbiome is causally associated with PsA at the genetic level and offer valuable insights into the etiology of PsA.

**Methods:**

In this study, we employed a bidirectional two-sample Mendelian randomization (MR) analysis to investigate the causal effects of the gut microbiome on PsA. Publicly accessible genome-wide association study summary data of gut microbiome were obtained from the MiBioGen consortium (*n* = 14,306), while the summary statistics of psoriatic arthropathies were sourced from the FinnGen consortium R8 release data (2,776 cases and 221,323 controls). The primary analytical method employed was inverse variance weighted (IVW), complemented by supplementary methods including MR-Egger, weighted median, weighted mode, maximum likelihood, MR-PRESSO, and cML-MA. Reverse MR analysis was performed on the bacteria that were found to be causally associated with PsA in forward MR analysis. Cochran’s IVW Q statistic was utilized to assess the heterogeneity of instrumental variables among the selected single nucleotide polymorphisms.

**Results:**

IVW estimates revealed that *Ruminococcaceae_UCG-002* (odds ratio (OR) = 0.792, 95% confidence interval (CI), 0.643–0.977, *p* = 0.029) exhibited a protective effect on PsA. Conversely, *Blautia* (OR = 1.362, 95% CI, 1.008–1.842, *p* = 0.044), *Eubacterium_fissicatena_group* (OR = 1.28, 95% CI, 1.075–1.524, *p* = 0.006), and *Methanobrevibacter* (OR = 1.31, 95% CI, 1.059–1.621, *p* = 0.013) showed a positive correlation with the risk of PsA. No significant heterogeneity, horizontal pleiotropy, or outliers were observed, and the results of the MR analysis remained unaffected by any single nucleotide polymorphisms. According to the results of reverse MR analysis, no significant causal effect of PsA was found on gut microbiome.

**Conclusion:**

This study establishes for the first time a causal relationship between the gut microbiome and PsA, providing potential valuable strategies for the prevention and treatment of PsA. Further randomized controlled trials are urgently warranted to support the targeted protective mechanisms of probiotics on PsA.

## Introduction

Psoriatic arthritis (PsA) is a chronic, immune-mediated, inflammatory arthropathy that clinically manifests with joint pain, stiffness, and swelling, making it one of the most severe comorbidities associated with psoriasis (Veale and Fearon, 2018). Once diagnosed with psoriasis, approximately one-third of patients will eventually transition to having PsA (Scher et al., 2019). Several potential risk factors have been identified for this transition (FitzGerald et al., 2021), including genetic susceptibility within the HLA region, comorbidities such as hyperlipidemia and obesity, variants in genes involved in interferon signaling and NF-κB signaling, and psoriasis-related factors such as psoriasis severity, potentially psoriasis location, and nail dystrophic changes (Veale and Fearon, 2018; Scher et al., 2019; FitzGerald et al., 2021). The prevalence of PsA worldwide ranges from 0.1 to 1% in the general population, with an estimated 520,000 cases in the US alone (Gelfand et al., 2005; Karmacharya et al., 2021). Despite some previous reports resuming a relatively mild course for most PsA patients, impaired function and reduced quality of life are the inevitable challenges they face. More importantly, PsA not only increases the risk of disability (Scher et al., 2019) but also contributes to the severity of morbidity and mortality associated with psoriasis (Gladman et al., 2005). Consequently, actively investigating the causes of PsA to provide novel and effective treatment strategies is of utmost importance.

In recent years, the notion that the gut microbiome significantly influences immune-mediated diseases like systemic lupus erythematosus, rheumatoid arthritis, ankylosing spondylitis, and multiple sclerosis has gained widespread acceptance (Zhou et al., 2020; Chen et al., 2022; Correale et al., 2022; Zhao et al., 2022). The gut microbiome primarily participates in nutrient processing, immune system development, colonization resistance, and the stimulation of various host activities, all vital for maintaining the body’s dynamic balance and human health (Adak and Khan, 2019). Furthermore, the gut microbiome not only modulates the mucosal and systemic immune systems but also exerts extra-intestinal effects at distant human body sites (Konig, 2020). While several observational studies have investigated the composition of the gut microbiome in patients with psoriasis, research on PsA remains limited (Myers et al., 2019). Scher et al. found that the altered gut microbiome of patients with PsA is characterized by decreased bacterial diversity as compared to a healthy fecal microbiome (Scher et al., 2015). More notably, they observed a decrease in the genera *Akkermansia, Ruminococcus, Pseudobutyrivibrio, Parabacteroides, Alistipes, and Coprococcus* in the PsA group compared to the healthy cohort. However, whether there is a causal relationship between the gut microbiome and PsA remains uncertain.

In traditional epidemiological studies, the inference of causality between exposure and outcome is greatly hindered by the existence of confounders that cannot be entirely eliminated, even in the presence of strong statistical associations. Mendelian randomization (MR) offers an innovative approach to integrate summary data from genome-wide association study (GWAS) and minimize the impact of confounders (Sekula et al., 2016). Due to the random allocation of genotypes from parents to offspring, the relationship between genetic variants and outcomes remains unaffected by common confounders (Bowden and Holmes, 2019). Previous MR studies have successfully identified several risk factors for PsA (Jin et al., 2023), such as the association between body mass index and PsA (Zhao et al., 2023), and the causal effect of inflammatory bowel disease on PsA (Freuer et al., 2022). Conversely, a higher physiological IL-17 level is linked to a decreased risk of PsA (Wu et al., 2021), and IL-12B provides protection against both psoriasis and PsA (Ek et al., 2021), as indicated by MR studies. Moreover, MR analysis demonstrated that the level of estimated bone mineral density does not have a causal effect on psoriasis and PsA (Xia et al., 2020). In the present study, we first reported the genetic association between microbiome and PsA, utilizing the summary-statistic GWAS data from the MiBioGen and FinnGen consortiums for further investigation.

## Methods

### Study design

**Figure 1:**
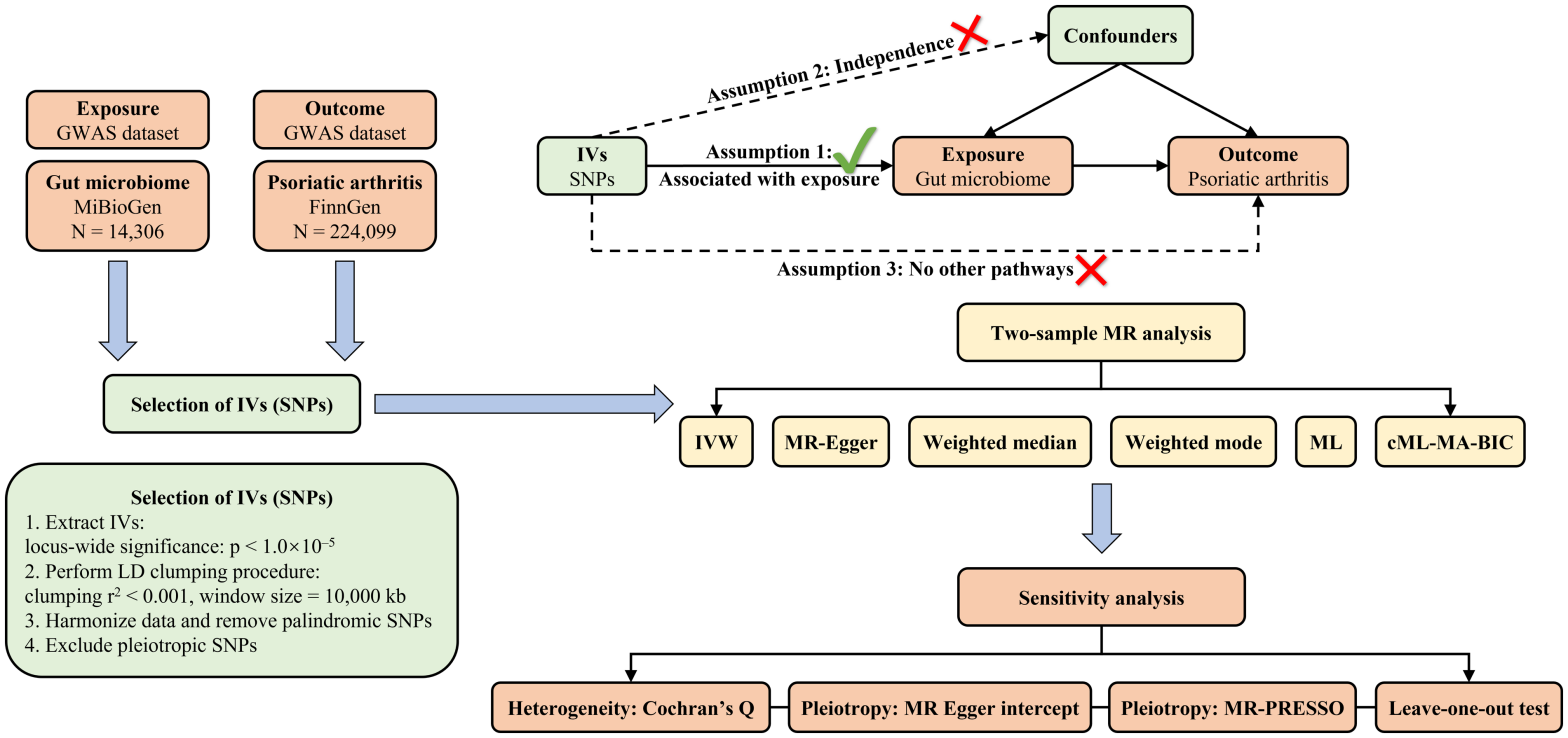
The diagrammatic description of the flow chart in MR analysis. A whole workflow of the MR analysis was manifested in this figure.

As depicted in Figure 1, the objective of this study was to select eligible instrumental variables (IVs) for MR analysis and investigate the causality based on the GWAS summary data of gut microbiome and PsA. To minimize the effect of bias on results, this study strictly adhered to three crucial assumptions of MR analysis: (1) the selected IVs were strongly associated with exposure; (2) the IVs were independent of any confounders; and (3) the IVs could only affect the outcome through the exposure.

### Data sources

GWAS summary statistics for gut microbiome were obtained from the MiBioGen consortium (https://mibiogen.gcc.rug.nl/. Accessed on March 12, 2023), which conducted the most comprehensive genome-wide meta-analysis published to date (van der Velde et al., 2019; Kurilshikov et al., 2021). To explore the relationship between autosomal human genetic variants and gut microbiome, the study included 24 population-based cohorts from various countries, such as the United States, Canada, Denmark, the United Kingdom, and so on. A total of 18,340 individuals, mostly of European descent (*n* = 14,306), participated in the study and provided informed consent. 119 bacterial taxa were identified in the current study for analysis, excluding 12 unknown genera (Kurilshikov et al., 2021). The data on PsA were obtained from the FinnGen consortium R8 (https://r8.finngen.fi/. Accessed on March 12, 2023) (Kurki et al., 2023). The study employed the phenotype “Psoriatic arthropathies” and included 223,099 Finnish adults, comprising 2,776 cases and 221,323 controls. All participants were of European ancestry and provided ethical permission and written informed consent. More detailed information about the participants, genotyping, imputation, and quality control, could be found on the FinnGen website (https://finngen.gitbook.io/documentation/. Accessed on March 12, 2023).

### Instrumental variables selection

To ensure the accuracy and validity of the conclusions regarding the causal association between gut microbiome and PsA, several criteria were employed for selecting optimal IVs: (1) single nucleotide polymorphisms (SNPs) significantly associated with each genus, meeting the locus-wide significance threshold of *p* < 1.0 × 10^−5^, were considered potential IVs (Burgess and Thompson, 2015; Sanna et al., 2019; Liu et al., 2022); (2) linkage disequilibrium (LD) between the included SNPs was eliminated since the presence of strong LD could result in biased results (*r^2^* < 0.001, clumping distance = 10,000 kb); (3) palindromic SNPs were excluded from the IVs to ensure that the effects of SNPs on exposure and outcome corresponded to the same allele; and (4) when the outcome GWAS did not include SNPs associated with exposure, the proxy SNPs remarkably related to the variants of interest were selected (*r^2^* > 0.8).

### Statistical analysis

In the current study, a two-sample MR analysis of the gut microbiome and PsA was performed through the TwoSampleMR (version 0.5.6) (Hemani et al., 2017), MR-PRESSO (version 1.0) (Verbanck et al., 2018), and cML-MA (Xue et al., 2021) packages in R version 4.2.1 (R Foundation for Statistical Computing, Vienna, Austria). The MR analysis was carried out in seven different ways: the random-effects inverse variance weighted (IVW) as the primary approach, and maximum likelihood (ML), MR Egger, weighted median, weighted mode, MR-PRESSO, and cML-MA as supplementary methods. The IVW method essentially employed a meta-analysis approach, utilizing weighted linear regression, to obtain an overall estimate of the effect of the gut microbiome on the risk of PsA. When there was no horizontal pleiotropy between SNPs, the IVW results could be accurate and unbiased by avoiding the impact of confounders (Burgess et al., 2016). Similarly, the ML method assumed the absence of horizontal pleiotropy and heterogeneity. Once these assumptions were established, the results would also be unbiased and the standard errors would be lower than IVW (Pierce and Burgess, 2013). The MR-Egger method assessed the average pleiotropy of IVs with the intercept term when pleiotropy was present on IVs. If the intercept term was zero, the results of the MR-Egger regression would be in accordance with IVW (Bowden et al., 2015). MR-Egger might lead to incorrect estimates due to the strong influence of external genetic variables. The weighted median could provide accurate estimates of causal effects even when at least 50% of IVs were invalid. As to mode evaluation, the weighted mode was more susceptible to the challenges in data collection (Hartwig et al., 2017). Given the low reliability and validity of statistical power in MR-Egger regression, MR pleiotropy residual sum and outlier (MR-PRESSO) method was commonly performed to identify significant outliers reflecting likely pleiotropic biases and reduce horizontal pleiotropy (Verbanck et al., 2018). Notably, the outliers identified during the distortion test of MR-PRESSO were excluded from the analysis, and the causal association was reassessed. Additionally, the constrained maximum likelihood and model average (CML-MA) method was employed to control correlated and uncorrelated pleiotropic effects (Xue et al., 2021). Cochran’s IVW Q statistics were performed to quantify the heterogeneity among the IVs based on the selected SNPs, with *p* > 0.05 indicating no heterogeneity (Hemani et al., 2018). In order to verify the reliability and stability of the causal effect estimates, a leave-one-out sensitivity analysis was conducted to identify potentially influential SNPs. Additionally, in order to evaluate the causal relationship between the gut microbiome and PsA, we conducted reverse MR analysis on the bacteria that were identified as causally linked to PsA in the forward MR analysis. The methodologies and parameters employed in this reverse MR analysis were consistent with those utilized in the forward MR analysis.

## Results

### Instrumental variables selection

After screening for SNPs related to exposure and eliminating strong LD effects, a total of 1,269 SNPs (locus-wide significance level, *p* < 1 × 10^−5^) were initially identified for 119 bacterial genera according to the selection criteria of IVs. Details of the selected IVs were given in Supplementary Table S1. The key information of these SNPs, including beta, standard error, odds ratio (OR), 95% confidence interval (CI), *p*-value, and *F* statistic, was systematically collected for further analysis.

### Two-sample MR analysis

As shown in Table 1 and Supplementary Table S2, ten bacterial genera found to be associated with PsA in at least one MR method were as follows: *Blautia*, *Butyricicoccus*, *Christensenellaceae_R-7_group*, *Defluviitaleaceae_UCG-011*, *Eubacterium_fissicatena_group*, *Family_XIII_AD3011_group*, *Fusicatenibacter*, *Methanobrevibacter*, *Oscillospira*, and *Ruminococcaceae_UCG-002*. Notably, IVW, ML, and cML-MA estimate all demonstrated that *Blautia* (IVW OR = 1.362, 95% CI, 1.008–1.842, *p* = 0.044; ML OR = 1.375, 95% CI, 1.011–1.869, *p* = 0.042; cML-MA OR = 1.369, 95% CI, 1.008–1.842, *p* = 0.044), *Eubacterium_fissicatena_group* (IVW OR = 1.28, 95% CI, 1.075–1.524, *p* = 0.006; ML OR = 1.293, 95% CI, 1.077–1.553, *p* = 0.006; cML-MA OR = 1.283, 95% CI, 1.063–1.55, *p* = 0.01), and *Methanobrevibacter* (IVW OR = 1.31, 95% CI, 1.059–1.621, *p* = 0.013; ML OR = 1.315, 95% CI, 1.055–1.639, *p* = 0.015; cML-MA OR = 1.311, 95% CI, 1.049–1.638, *p* = 0.017) were risk factors for PsA, while the results of IVW and ML estimates showed that *Ruminococcaceae_UCG-002* (IVW OR = 0.792, 95% CI, 0.643–0.977, *p* = 0.029; ML OR = 0.794, 95% CI, 0.646–0.975, *p* = 0.028) had a protective effect on PsA. Although IVW results did not support the causal associations of *Butyricicoccus*, *Christensenellaceae_R-7_group*, *Defluviitaleaceae_UCG-011*, and *Family_XIII_AD3011_group* (*p* > 0.05), the ML estimates represented that *Butyricicoccus* (OR = 0.651, 95% CI: 0.454–0.933, *p* = 0.019), *Defluviitaleaceae_UCG-011* (OR = 1.34, 95% CI: 1.026–1.749, *p* = 0.032), and *Family_XIII_AD3011_group* (OR = 1.338, 95% CI: 1.016–1.764, *p* = 0.038) were causally correlated with PsA. Similarly, the cML-MA estimates of *Christensenellaceae_R-7_group* (OR = 0.663, 95% CI: 0.461–0.955, *p* = 0.027) suggested a causal relationship with PsA. The MR estimates of weighted median suggested that *Defluviitaleaceae_UCG-011* (OR = 1.497, 95% CI, 1.031–2.174, *p* = 0.034) and *Fusicatenibacter* (OR = 1.416, 95% CI, 1.004–1.997, *p* = 0.047) were positively correlated with the risk of PsA and *Christensenellaceae_R-7_group* (OR = 0.613, 95% CI, 0.397–0.948, *p* = 0.028) were negatively correlated with PsA risk. Moreover, the results of MR Egger indicated that *Oscillospira* (OR = 0.151, 95% CI, 0.046–0.491, *p* = 0.02) showed its suggestive protective effect against PsA.

**Table 1:** MR estimates for the association between gut microbiome and PsA.

Bacterial taxa (exposure)	MR methods	No. of SNP	Beta	SE	OR	95% CI	*p*-value	*F* statistic
*Blautia*	IVW	13	0.309	0.154	1.362	1.008–1.842	0.044	25.6
	MR Egger	13	0.306	0.406	1.358	0.613–3.008	0.467	
	Weighted median	13	0.373	0.211	1.452	0.96–2.196	0.078	
	Weighted mode	13	0.498	0.383	1.645	0.776–3.484	0.218	
	Maximum likelihood	13	0.318	0.157	1.375	1.011–1.869	0.042	
	cML-MA-BIC	13	0.314	0.159	1.369	1.002–1.87	0.049	
*Butyricicoccus*	IVW	8	−0.39	0.218	0.677	0.442–1.038	0.073	39.96
	MR Egger	8	0.331	0.325	1.392	0.736–2.635	0.348	
	Weighted median	8	−0.054	0.233	0.947	0.6–1.496	0.816	
	Weighted mode	8	−0.045	0.266	0.956	0.568–1.61	0.87	
	Maximum likelihood	8	−0.43	0.184	0.651	0.454–0.933	0.019	
	cML-MA-BIC	8	−0.306	0.202	0.736	0.495–1.095	0.13	
*Christensenellaceae_R-7_group*	IVW	10	−0.162	0.23	0.85	0.541–1.335	0.481	21.86
	MR Egger	10	0.026	0.751	1.026	0.236–4.47	0.973	
	Weighted median	10	−0.489	0.222	0.613	0.397–0.948	0.028	
	Weighted mode	10	−0.531	0.296	0.588	0.329–1.051	0.106	
	Maximum likelihood	10	−0.179	0.178	0.837	0.59–1.187	0.317	
	cML-MA-BIC	10	−0.41	0.186	0.663	0.461–0.955	0.027	
*Defluviitaleaceae_UCG-011*	IVW	9	0.272	0.151	1.312	0.976–1.765	0.072	24.14
	MR Egger	9	−0.433	0.514	0.649	0.237–1.775	0.427	
	Weighted median	9	0.404	0.19	1.497	1.031–2.174	0.034	
	Weighted mode	9	0.435	0.332	1.544	0.805–2.962	0.227	
	Maximum likelihood	9	0.292	0.136	1.34	1.026–1.749	0.032	
	cML-MA-BIC	9	0.278	0.146	1.321	0.992–1.76	0.057	
*Eubacterium_fissicatena_group*	IVW	9	0.247	0.089	1.28	1.075–1.524	0.006	22.39
	MR Egger	9	−0.43	0.463	0.65	0.262–1.613	0.384	
	Weighted median	9	0.212	0.125	1.237	0.968–1.579	0.089	
	Weighted mode	9	0.211	0.176	1.235	0.875–1.743	0.264	
	Maximum likelihood	9	0.257	0.093	1.293	1.077–1.553	0.006	
	cML-MA-BIC	9	0.249	0.096	1.283	1.063–1.55	0.01	
*Family_XIII_AD3011_group*	IVW	13	0.274	0.17	1.316	0.942–1.837	0.107	22.83
	MR Egger	13	−0.543	0.802	0.581	0.121–2.798	0.512	
	Weighted median	13	0.247	0.202	1.28	0.862–1.903	0.221	
	Weighted mode	13	0.27	0.317	1.309	0.704–2.437	0.411	
	Maximum likelihood	13	0.291	0.141	1.338	1.016–1.764	0.038	
	cML-MA-BIC	13	0.166	0.159	1.18	0.863–1.613	0.299	
*Fusicatenibacter*	IVW	18	0.227	0.128	1.254	0.975–1.613	0.077	23.33
	MR Egger	18	−0.064	0.481	0.938	0.365–2.41	0.896	
	Weighted median	18	0.348	0.175	1.416	1.004–1.997	0.047	
	Weighted mode	18	0.528	0.321	1.696	0.903–3.183	0.119	
	Maximum likelihood	18	0.233	0.129	1.262	0.979–1.626	0.072	
	cML-MA-BIC	18	0.237	0.131	1.267	0.98–1.639	0.071	
*Methanobrevibacter*	IVW	6	0.27	0.109	1.31	1.059–1.621	0.013	22
	MR Egger	6	0.607	0.407	1.836	0.827–4.075	0.21	
	Weighted median	6	0.235	0.148	1.265	0.946–1.693	0.113	
	Weighted mode	6	0.229	0.165	1.257	0.91–1.737	0.224	
	Maximum likelihood	6	0.274	0.112	1.315	1.055–1.639	0.015	
	cML-MA-BIC	6	0.271	0.114	1.311	1.049–1.638	0.017	
*Oscillospira*	IVW	8	−0.223	0.189	0.8	0.552–1.158	0.237	21.58
	MR Egger	8	−1.893	0.603	0.151	0.046–0.491	0.02	
	Weighted median	8	−0.359	0.208	0.699	0.465–1.05	0.085	
	Weighted mode	8	−0.465	0.328	0.628	0.33–1.194	0.199	
	Maximum likelihood	8	−0.24	0.149	0.787	0.588–1.053	0.107	
	cML-MA-BIC	8	−0.274	0.163	0.76	0.552–1.047	0.094	
*Ruminococcaceae_UCG-002*	IVW	22	−0.233	0.107	0.792	0.643–0.977	0.029	26.96
	MR Egger	22	−0.316	0.287	0.729	0.416–1.28	0.284	
	Weighted median	22	−0.167	0.152	0.846	0.628–1.139	0.271	
	Weighted mode	22	−0.142	0.243	0.867	0.539–1.397	0.565	
	Maximum likelihood	22	−0.231	0.105	0.794	0.646–0.975	0.028	
	cML-MA-BIC	22	−0.18	0.111	0.835	0.672–1.039	0.106	

MR, Mendelian randomization; PsA, psoriatic arthritis; SNP, single nucleotide polymorphism; SE, standard error; OR, odds ratio; CI, confidence interval; IVW, inverse variance weighted; ML, maximum likelihood.

**Figure 2:**
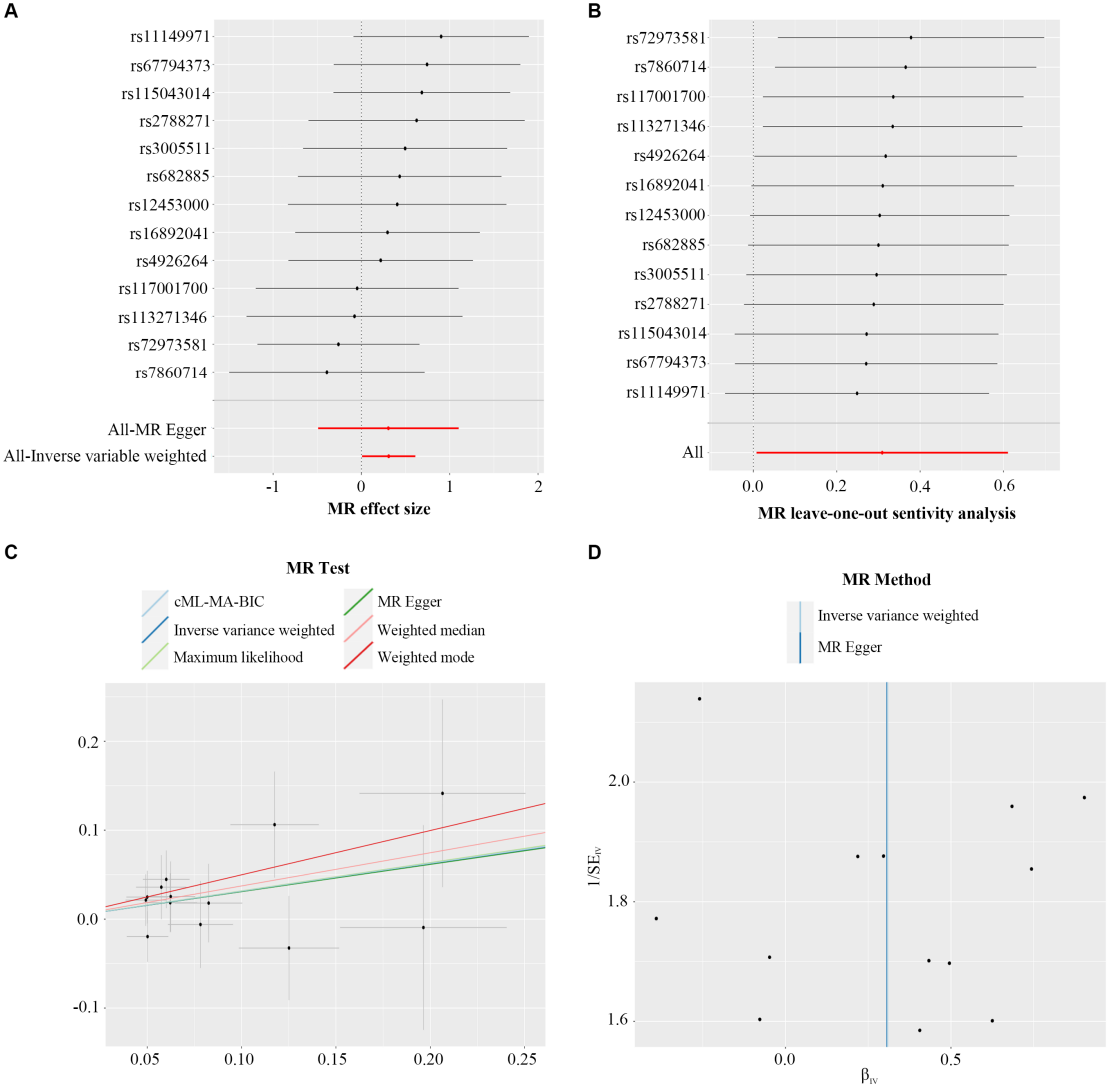
Forest plot **(A)**, sensitivity analysis **(B)**, scatter plot **(C)**, and funnel plot **(D)** of the causal effect of *Blautia* on PsA risk.

MR-Egger regression intercept analysis was used to assess the horizontal pleiotropy between IVs and outcomes, as shown in Supplementary Table S3, the results showed that there existed significant horizontal pleiotropy between *Butyricicoccus* (*p* = 0.042) and *Oscillospira* (*p* = 0.029) while there was no evidence of directional horizontal pleiotropy in other IVs (*p* > 0.05). No significant outliers were found in the analysis of *Blautia* (global test *p* = 0.876), *Butyricicoccus* (global test *p* = 0.142), *Christensenellaceae_R-7_group* (global test *p* = 0.062), *Defluviitaleaceae_UCG-011* (global test *p* = 0.204), *Eubacterium_fissicatena_group* (global test *p* = 0.495), *Family_XIII_AD3011_group* (global test *p* = 0.099), *Fusicatenibacter* (global test *p* = 0.906), *Methanobrevibacter* (global test *p* = 0.899), *Oscillospira* (global test *p* = 0.112), and *Ruminococcaceae_UCG-002* (global test *p* = 0.411) by MR-PRESSO (Supplementary Table S4). Among these ten causal associations, the *F*-statistics of the IVs ranged from 21.58 to 39.96, indicating that there was no weak IVs bias (Table 1). The results of Cochran’s Q statistic indicated no significant heterogeneity in PsA IVs (*p* > 0.05, Supplementary Table S5). According to the results of reverse MR analysis, a notable absence of a causal relationship was observed between PsA and gut microbiome (Supplementary Table S6). Therefore, the two-sample MR estimates found that *Blautia* (Figure 2), *Defluviitaleaceae_UCG-011* (Supplementary Figure S1), *Eubacterium_fissicatena_group* (Supplementary Figure S2), *Family_XIII_AD3011_group* (Supplementary Figure S3), *Fusicatenibacter* (Supplementary Figure S4), and *Methanobrevibacter* (Supplementary Figure S5) were positively related to PsA risk, and *Butyricicoccus* (Supplementary Figure S6), *Christensenellaceae_R-7_group* (Supplementary Figure S7), *Oscillospira* (Supplementary Figure S8), and *Ruminococcaceae_UCG-002* (Supplementary Figure S9), played protective roles in the pathogenesis of PsA.

## Discussion

In this study, a two-sample MR analysis was performed to assess the genetic causal association between gut microbiome and PsA. The findings indicated that *Blautia*, *Defluviitaleaceae_UCG-011*, *Eubacterium_fissicatena_group*, *Family_XIII_AD3011_group*, *Fusicatenibacter*, and *Methanobrevibacter* might serve as risk factors for the onset of PsA, while *Butyricoccus*, *Christensenellaceae_R-7_group*, *Oscillospira* and *Ruminococcaceae_UCG-002* showed significant protective effects against PsA.

PsA is one of the most common comorbidities observed in patients with psoriasis. Although the exact etiology has still not been fully investigated, it is well known that genetic, immunological, and environmental factors all play crucial roles in the development of PsA (Veale and Fearon, 2018). In terms of genetic susceptibility and Th17-predominant immune activity (Vlachos et al., 2016), similar pathogenesis is also observed in other immune-mediated inflammatory diseases, such as inflammatory bowel disease, which is one of the characteristic extra-articular manifestations of PsA (Lloyd-Price et al., 2019). The roles of the gut microbiome in the development of inflammatory bowel disease have been extensively supported (Sartor and Wu, 2017). The intestine, usually considered a key target organ in axial spondyloarthritis, is likely to be a site of immunological changes that initiate the disease. Several published articles have reported a gut-joint axis in spondyloarthritis (Gilis et al., 2018), and a similar link may probably exist in PsA. Interestingly, joints were previously considered as a sterile environment whereas bacterial components have now been found to exist in the joints of rheumatoid arthritis patients (Kempsell et al., 2000). These components may reach the joints either via circulation or through the intestinal lymphatic system (Berlinberg and Kuhn, 2020), suggesting that intestinal dysbiosis can be manifested via migration from circulation to joints. Unlike the intestine, where pathogens and symbionts coexist in symbiosis, the joint does not possess its “own” microbiome (Scarpa et al., 2000). However, the impact of these microbiomes on PsA, whether beneficial or detrimental, requires further investigation.

Utilizing data from the FinnGen consortium R8, our study performed the MR analysis on a large cohort of 223,099 Finnish adults to conclude that ten gut microbial taxa were associated with PsA. Through the use of genetic IVs, the gut microbiome and the onset of PsA were demonstrated to be causal. In a recent observational study, noticeable differences had been reported in the overall abundance of the gut microbiome in individuals with PsA compared to normotensive individuals (Scher et al., 2015). When compared to healthy fecal microbiomes, Scher et al. (2015) highlighted a decrease in overall diversity in PsA fecal microbiomes. Additionally, they also reported a decrease in the genera *Ruminococcus* within PsA patients versus the healthy cohort. In line with the present study, *Ruminococcaceae_UCG-002* was found to have a negative association with PsA risk. The protective effect of *Ruminococcus* in reducing low-grade inflammation partly contributed to its production of butyrate, which was not only the primary energy source for intestinal bacteria but also the beneficial healthy factor to cause decreased intestinal permeability (Milani et al., 2017). Short-chain fatty acids (SCFAs), mostly consisting of acetic acid, propionic acid, and butyric acid, were proven to promote intestinal health and create a resilient environment resistant to pathogenic bacteria (Smith et al., 2013). In the present study, part of the gut microbiome identified to be related to PsA were SCFAs-producing bacteria, including *Butyricoccus* (Bojović et al., 2020), *ChristensenellaceaeR.7* (Jian et al., 2022), *Oscillospira* (Konig, 2020), *Ruminococcaceae_UCG-002* (Smith et al., 2013; Jiao et al., 2018), *Blautia*, and *Fusicatenibacter* (Nishiwaki et al., 2022). As a significant SCFA, butyrate was observed to have important roles in anti-inflammation (Vinolo et al., 2011), oxidative stress reduction (Lopez-Siles et al., 2012), and integrity of intestinal epithelial barrier maintenance (Plöger et al., 2012). It implied that the adverse losses of butyrate-producing bacteria might play a vital role in PsA pathogenesis by allowing local inflammatory response that in turn impaired the function of the gut epithelial barrier and compromised its role in regulating the presentation of gastrointestinal antigens to immune cells and systemic circulation (Bischoff et al., 2014). Moreover, dysbiosis in PsA, which allowed for bacterial translocation to extraintestinal sites including the joints, was also proposed as a model for PsA.

Contrary to previous studies, our findings revealed that *Blautia* and *Fusicatenibacter*, commonly considered probiotics, were actually risk factors for PsA at the genus level. These inconsistent results highlighted the considerable inter-and intra-species diversity that could impact host health. Therefore, the establishment of a standardized and detailed gut microbiome classification system is crucial for further mechanism research and clinical guidance. *Methanobrevibacter*, a genus of archaeal anaerobes and methanogens, has the ability to convert hydrogen gas into methane. Methanogens were commonly found in the gastrointestinal tract of animals (Janssen and Kirs, 2008) and provided protection against organ injury through their anti-apoptotic, anti-oxidative, and anti-inflammatory actions (Wang et al., 2021). Recently, several metagenomics studies have suggested that methane production of *Methanobrevibacter* might be closely connected with the etiology of digestive tract diseases in humans (Triantafyllou et al., 2014; Hoegenauer et al., 2022). A pilot study found that the abundance of *Family_XIII_AD3011_Group*, an intestinal probiotic, was significantly lower in the PsA group than in the NO PsA group (Lin et al., 2022). However, our findings suggested that the genus *Family_XIII_AD3011_Group* was positively linked to PsA risk. There was relatively little previous research on *Eubacterium*, which was crucial in the circulation of bile acids (Song et al., 2019). Notably, *Eubacterium_fissicatena_group* was reported to be linked with obesity-related metabolic disorders and intestinal inflammation (Nagayama et al., 2020; Song et al., 2021). These findings demonstrated that gut injury might participate in PsA progression, which was attributed to its decreased ability to produce butyric acid (Li et al., 2021). Although there is scarce evidence from available research on the relationships between the microbiome and PsA, it is undeniable that PsA patients often experience gut microbiome dysbiosis. All of the aforementioned results indicate that they are in a tight causal connection, and further research on their specific mechanisms is highly warranted. In general, three different approaches to manipulating the microbiome have been identified: (1) direct modulation by adding or eliminating specific bacterial strains or communities; (2) indirect modulation through diet, prebiotic medication, or other environmental factors; or (3) replacement of the indigenous microbiome through fecal microbiome transplantation (FMT) from a suitable donor (Miyauchi et al., 2023). In addition, given the potential for personalized treatment approaches based on an individual’s gut microbiome profile. Tailoring therapies to a patient’s unique microbial composition can be taken into consideration as well.

The main strength of this study lay in minimizing bias resulting from confounders through MR analysis, enhancing its credibility compared to conventional observational studies. Notably, this study was the first MR analysis to explore the causal association between gut microbiome and PsA. Nevertheless, several limitations were also worthy of consideration. To start with, the overwhelming majority of participants in the gut microbiome GWAS meta-analysis were of European descent in the MR analysis, limiting the extrapolation of the findings to other ethnic groups. Thus, the results of this study might not be entirely suitable for individuals of non-European descent. Secondly, gender was not restricted in this study. It was therefore of great necessity to consider whether there was a difference when applied to the male or female population alone. Thirdly, given that applying a rigorous multiple-testing correction might be overly conservative, which would overlook potential strains that were causally associated with PsA, multiple tests were not taken into account. In addition, it was impossible to conduct subgroup analysis, such as distinguishing between early-onset and late-onset PsA, due to the use of summary data on PsA rather than raw data in this study. Finally, the lowest taxonomic class was at the genetic level, this limitation prevented us from deeply exploring whether there existed a causal relationship between gut microbiome and PsA.

## Conclusion

In summary, this comprehensive bidirectional two-sample MR analysis provided compelling evidence for a causal association between the gut microbiome and PsA. Several types of gut microbiomes identified in this study might offer valuable insights into the prevention and treatment of PsA, as well as strategies for PsA pathogenesis.

## Data availability statement

The original contributions presented in the study are included in the article/Supplementary material, further inquiries can be directed to the corresponding author.

## Ethics statement

The studies involving humans were approved by Shanghai Changhai Hospital ethics committee. The studies were conducted in accordance with the local legislation and institutional requirements. Written informed consent for participation was not required from the participants or the participants’ legal guardians/next of kin in accordance with the national legislation and institutional requirements. The manuscript presents research on animals that do not require ethical approval for their study.

## Author contributions

XQ: Conceptualization, Formal analysis, Investigation, Validation, Visualization, Writing – original draft, Writing – review & editing. ZF: Data curation, Methodology, Software, Supervision, Writing – review & editing. CD: Supervision, Validation, Writing – review & editing. WZ: Validation, Writing – review & editing. WT: Visualization, Writing – review & editing. JH: Supervision, Writing – review & editing. SZ: Resources, Writing – review & editing. DZ: Data curation, Funding acquisition, Supervision, Writing – original draft, Writing – review & editing.
