# Supplementary material for: Genetic causal relationship between gut microbiome and psoriatic arthritis: a bidirectional two-sample Mendelian randomization study
Source: Front Microbiol. 2023 Oct 31;14:1265786. doi: 10.3389/fmicb.2023.1265786 (PMC10644104; doi:10.3389/fmicb.2023.1265786)
Supplement: Supplementary file 1 [file Data_Sheet_1.DOCX]

**Legends for supplementary materials:**

**Supplemental Table S1:** Full results of MR estimates for the association between gut microbiome and PsA.

| **Table S1 Full results of MR estimates for the association between gut microbiome and PsA.** | | | | | | | |
| --- | --- | --- | --- | --- | --- | --- | --- |
| **Bacterial taxa (exposure)** | **MR methods** | **No. of SNP** | **Beta** | **SE** | **OR** | **95% CI** | ***P*-value** |
| *Actinomyces* | Inverse variance weighted | 7 | -0.125 | 0.127 | 0.882 | 0.688-1.13 | 0.321 |
|  | MR Egger | 7 | -0.283 | 0.309 | 0.754 | 0.411-1.382 | 0.403 |
|  | Weighted median | 7 | -0.109 | 0.154 | 0.897 | 0.663-1.213 | 0.479 |
|  | Weighted mode | 7 | -0.12 | 0.193 | 0.887 | 0.608-1.295 | 0.558 |
|  | Maximum likelihood | 7 | -0.127 | 0.128 | 0.881 | 0.685-1.133 | 0.322 |
|  | cML-MA-BIC | 7 | -0.117 | 0.131 | 0.889 | 0.687-1.151 | 0.372 |
| *Adlercreutzia* | Inverse variance weighted | 8 | 0.186 | 0.135 | 1.204 | 0.925-1.569 | 0.168 |
|  | MR Egger | 8 | -0.34 | 0.603 | 0.712 | 0.218-2.321 | 0.593 |
|  | Weighted median | 8 | 0.262 | 0.172 | 1.3 | 0.929-1.82 | 0.126 |
|  | Weighted mode | 8 | 0.344 | 0.266 | 1.411 | 0.837-2.378 | 0.238 |
|  | Maximum likelihood | 8 | 0.193 | 0.137 | 1.212 | 0.927-1.586 | 0.16 |
|  | cML-MA-BIC | 8 | 0.196 | 0.139 | 1.216 | 0.926-1.597 | 0.16 |
| *Akkermansia* | Inverse variance weighted | 11 | 0.144 | 0.135 | 1.155 | 0.887-1.504 | 0.285 |
|  | MR Egger | 11 | 0.319 | 0.474 | 1.376 | 0.544-3.482 | 0.518 |
|  | Weighted median | 11 | 0.14 | 0.179 | 1.15 | 0.809-1.634 | 0.436 |
|  | Weighted mode | 11 | 0.102 | 0.273 | 1.107 | 0.649-1.889 | 0.717 |
|  | Maximum likelihood | 11 | 0.15 | 0.133 | 1.162 | 0.895-1.509 | 0.26 |
|  | cML-MA-BIC | 11 | 0.111 | 0.147 | 1.118 | 0.838-1.49 | 0.448 |
| *Alistipes* | Inverse variance weighted | 13 | 0.032 | 0.162 | 1.033 | 0.751-1.42 | 0.842 |
|  | MR Egger | 13 | -1.076 | 0.782 | 0.341 | 0.074-1.577 | 0.196 |
|  | Weighted median | 13 | 0.137 | 0.201 | 1.147 | 0.773-1.702 | 0.495 |
|  | Weighted mode | 13 | 0.196 | 0.355 | 1.217 | 0.607-2.441 | 0.591 |
|  | Maximum likelihood | 13 | 0.034 | 0.164 | 1.035 | 0.75-1.428 | 0.835 |
|  | cML-MA-BIC | 13 | 0.043 | 0.167 | 1.044 | 0.753-1.447 | 0.798 |
| *Allisonella* | Inverse variance weighted | 8 | 0.045 | 0.08 | 1.046 | 0.895-1.223 | 0.572 |
|  | MR Egger | 8 | 0.474 | 0.54 | 1.607 | 0.558-4.632 | 0.413 |
|  | Weighted median | 8 | 0.018 | 0.101 | 1.018 | 0.836-1.24 | 0.86 |
|  | Weighted mode | 8 | 0.02 | 0.148 | 1.02 | 0.763-1.364 | 0.897 |
|  | Maximum likelihood | 8 | 0.046 | 0.081 | 1.047 | 0.894-1.226 | 0.568 |
|  | cML-MA-BIC | 8 | 0.043 | 0.082 | 1.044 | 0.889-1.225 | 0.602 |
| *Alloprevotella* | Inverse variance weighted | 6 | -0.055 | 0.115 | 0.947 | 0.756-1.185 | 0.632 |
|  | MR Egger | 6 | 0.625 | 1.144 | 1.867 | 0.198-17.574 | 0.614 |
|  | Weighted median | 6 | -0.013 | 0.125 | 0.988 | 0.773-1.262 | 0.92 |
|  | Weighted mode | 6 | 0.114 | 0.219 | 1.121 | 0.73-1.721 | 0.624 |
|  | Maximum likelihood | 6 | -0.058 | 0.096 | 0.943 | 0.782-1.138 | 0.541 |
|  | cML-MA-BIC | 6 | -0.047 | 0.101 | 0.954 | 0.783-1.162 | 0.638 |
| *Anaerofilum* | Inverse variance weighted | 10 | -0.067 | 0.115 | 0.936 | 0.747-1.172 | 0.563 |
|  | MR Egger | 10 | -0.739 | 0.618 | 0.478 | 0.142-1.603 | 0.266 |
|  | Weighted median | 10 | -0.01 | 0.133 | 0.99 | 0.762-1.286 | 0.939 |
|  | Weighted mode | 10 | -0.058 | 0.23 | 0.943 | 0.601-1.481 | 0.806 |
|  | Maximum likelihood | 10 | -0.071 | 0.095 | 0.931 | 0.772-1.123 | 0.454 |
|  | cML-MA-BIC | 10 | -0.097 | 0.106 | 0.908 | 0.738-1.117 | 0.36 |
| *Anaerostipes* | Inverse variance weighted | 13 | 0.159 | 0.151 | 1.172 | 0.872-1.576 | 0.293 |
|  | MR Egger | 13 | -0.141 | 0.548 | 0.869 | 0.297-2.542 | 0.802 |
|  | Weighted median | 13 | 0.263 | 0.202 | 1.3 | 0.876-1.931 | 0.193 |
|  | Weighted mode | 13 | 0.452 | 0.341 | 1.571 | 0.805-3.066 | 0.21 |
|  | Maximum likelihood | 13 | 0.163 | 0.154 | 1.177 | 0.871-1.592 | 0.289 |
|  | cML-MA-BIC | 13 | 0.176 | 0.158 | 1.193 | 0.876-1.624 | 0.264 |
| *Anaerotruncus* | Inverse variance weighted | 13 | 0.073 | 0.15 | 1.076 | 0.801-1.445 | 0.626 |
|  | MR Egger | 13 | 0.316 | 0.452 | 1.371 | 0.565-3.329 | 0.5 |
|  | Weighted median | 13 | 0.011 | 0.21 | 1.011 | 0.671-1.524 | 0.959 |
|  | Weighted mode | 13 | -0.04 | 0.284 | 0.961 | 0.551-1.675 | 0.89 |
|  | Maximum likelihood | 13 | 0.078 | 0.148 | 1.081 | 0.808-1.445 | 0.601 |
|  | cML-MA-BIC | 13 | 0.074 | 0.154 | 1.077 | 0.797-1.455 | 0.63 |
| *Bacteroides* | Inverse variance weighted | 9 | 0.012 | 0.203 | 1.012 | 0.68-1.507 | 0.952 |
|  | MR Egger | 9 | -0.499 | 1.11 | 0.607 | 0.069-5.349 | 0.667 |
|  | Weighted median | 9 | -0.093 | 0.249 | 0.911 | 0.559-1.485 | 0.709 |
|  | Weighted mode | 9 | -0.131 | 0.378 | 0.878 | 0.418-1.841 | 0.739 |
|  | Maximum likelihood | 9 | 0.013 | 0.178 | 1.013 | 0.715-1.436 | 0.942 |
|  | cML-MA-BIC | 9 | -0.011 | 0.186 | 0.989 | 0.687-1.424 | 0.953 |
| *Barnesiella* | Inverse variance weighted | 14 | 0.073 | 0.128 | 1.075 | 0.837-1.382 | 0.57 |
|  | MR Egger | 14 | 0.835 | 0.484 | 2.306 | 0.893-5.953 | 0.11 |
|  | Weighted median | 14 | 0.037 | 0.169 | 1.038 | 0.746-1.445 | 0.825 |
|  | Weighted mode | 14 | 0.031 | 0.264 | 1.031 | 0.615-1.73 | 0.908 |
|  | Maximum likelihood | 14 | 0.075 | 0.13 | 1.078 | 0.836-1.39 | 0.563 |
|  | cML-MA-BIC | 14 | 0.067 | 0.131 | 1.069 | 0.826-1.383 | 0.612 |
| *Bifidobacterium* | Inverse variance weighted | 13 | 0.068 | 0.116 | 1.07 | 0.852-1.344 | 0.558 |
|  | MR Egger | 13 | -0.101 | 0.297 | 0.904 | 0.505-1.618 | 0.74 |
|  | Weighted median | 13 | -0.035 | 0.161 | 0.965 | 0.704-1.324 | 0.827 |
|  | Weighted mode | 13 | -0.108 | 0.197 | 0.898 | 0.61-1.321 | 0.594 |
|  | Maximum likelihood | 13 | 0.071 | 0.118 | 1.074 | 0.853-1.353 | 0.545 |
|  | cML-MA-BIC | 13 | 0.065 | 0.119 | 1.067 | 0.844-1.348 | 0.588 |
| *Bilophila* | Inverse variance weighted | 13 | 0.242 | 0.142 | 1.274 | 0.965-1.683 | 0.088 |
|  | MR Egger | 13 | -0.145 | 0.72 | 0.865 | 0.211-3.548 | 0.845 |
|  | Weighted median | 13 | 0.236 | 0.191 | 1.267 | 0.872-1.841 | 0.215 |
|  | Weighted mode | 13 | 0.118 | 0.304 | 1.125 | 0.62-2.039 | 0.705 |
|  | Maximum likelihood | 13 | 0.252 | 0.139 | 1.286 | 0.98-1.688 | 0.069 |
|  | cML-MA-BIC | 13 | 0.235 | 0.145 | 1.264 | 0.952-1.679 | 0.105 |
| *Blautia* | Inverse variance weighted | 13 | 0.309 | 0.154 | 1.362 | 1.008-1.842 | 0.044 |
|  | MR Egger | 13 | 0.306 | 0.406 | 1.358 | 0.613-3.008 | 0.467 |
|  | Weighted median | 13 | 0.373 | 0.211 | 1.452 | 0.96-2.196 | 0.078 |
|  | Weighted mode | 13 | 0.498 | 0.383 | 1.645 | 0.776-3.484 | 0.218 |
|  | Maximum likelihood | 13 | 0.318 | 0.157 | 1.375 | 1.011-1.869 | 0.042 |
|  | cML-MA-BIC | 13 | 0.314 | 0.159 | 1.369 | 1.002-1.87 | 0.049 |
| *Butyricicoccus* | Inverse variance weighted | 8 | -0.39 | 0.218 | 0.677 | 0.442-1.038 | 0.073 |
|  | MR Egger | 8 | 0.331 | 0.325 | 1.392 | 0.736-2.635 | 0.348 |
|  | Weighted median | 8 | -0.054 | 0.233 | 0.947 | 0.6-1.496 | 0.816 |
|  | Weighted mode | 8 | -0.045 | 0.266 | 0.956 | 0.568-1.61 | 0.87 |
|  | Maximum likelihood | 8 | -0.43 | 0.184 | 0.651 | 0.454-0.933 | 0.019 |
|  | cML-MA-BIC | 8 | -0.306 | 0.202 | 0.736 | 0.495-1.095 | 0.13 |
| *Butyricimonas* | Inverse variance weighted | 13 | -0.011 | 0.125 | 0.989 | 0.774-1.263 | 0.927 |
|  | MR Egger | 13 | 0.184 | 0.443 | 1.202 | 0.505-2.863 | 0.686 |
|  | Weighted median | 13 | -0.038 | 0.17 | 0.963 | 0.69-1.343 | 0.822 |
|  | Weighted mode | 13 | -0.049 | 0.271 | 0.952 | 0.56-1.618 | 0.858 |
|  | Maximum likelihood | 13 | -0.012 | 0.127 | 0.988 | 0.771-1.267 | 0.925 |
|  | cML-MA-BIC | 13 | -0.018 | 0.128 | 0.983 | 0.764-1.264 | 0.891 |
| *Butyrivibrio* | Inverse variance weighted | 15 | -0.031 | 0.077 | 0.97 | 0.833-1.129 | 0.694 |
|  | MR Egger | 15 | 0.284 | 0.334 | 1.329 | 0.69-2.559 | 0.41 |
|  | Weighted median | 15 | -0.01 | 0.089 | 0.99 | 0.831-1.18 | 0.911 |
|  | Weighted mode | 15 | 0.095 | 0.139 | 1.099 | 0.837-1.443 | 0.507 |
|  | Maximum likelihood | 15 | -0.031 | 0.063 | 0.97 | 0.858-1.097 | 0.625 |
|  | cML-MA-BIC | 15 | 0.011 | 0.073 | 1.011 | 0.877-1.167 | 0.876 |
| *CandidatusSoleaferrea* | Inverse variance weighted | 10 | -0.112 | 0.101 | 0.894 | 0.733-1.091 | 0.271 |
|  | MR Egger | 10 | 1.121 | 1.083 | 3.067 | 0.367-25.643 | 0.331 |
|  | Weighted median | 10 | -0.201 | 0.137 | 0.818 | 0.626-1.069 | 0.141 |
|  | Weighted mode | 10 | -0.217 | 0.223 | 0.805 | 0.52-1.246 | 0.357 |
|  | Maximum likelihood | 10 | -0.111 | 0.104 | 0.895 | 0.731-1.097 | 0.286 |
|  | cML-MA-BIC | 10 | -0.126 | 0.108 | 0.881 | 0.714-1.089 | 0.241 |
| *Catenibacterium* | Inverse variance weighted | 5 | 0.03 | 0.136 | 1.03 | 0.789-1.344 | 0.827 |
|  | MR Egger | 5 | 0.669 | 1.401 | 1.951 | 0.125-30.399 | 0.666 |
|  | Weighted median | 5 | 0.069 | 0.159 | 1.071 | 0.784-1.462 | 0.666 |
|  | Weighted mode | 5 | 0.006 | 0.216 | 1.006 | 0.658-1.537 | 0.981 |
|  | Maximum likelihood | 5 | 0.031 | 0.113 | 1.032 | 0.827-1.287 | 0.782 |
|  | cML-MA-BIC | 5 | 0.013 | 0.12 | 1.013 | 0.8-1.283 | 0.913 |
| *Christensenellaceae_R-7_group* | Inverse variance weighted | 10 | -0.162 | 0.23 | 0.85 | 0.541-1.335 | 0.481 |
|  | MR Egger | 10 | 0.026 | 0.751 | 1.026 | 0.236-4.47 | 0.973 |
|  | Weighted median | 10 | -0.489 | 0.222 | 0.613 | 0.397-0.948 | 0.028 |
|  | Weighted mode | 10 | -0.531 | 0.296 | 0.588 | 0.329-1.051 | 0.106 |
|  | Maximum likelihood | 10 | -0.179 | 0.178 | 0.837 | 0.59-1.187 | 0.317 |
|  | cML-MA-BIC | 10 | -0.41 | 0.186 | 0.663 | 0.461-0.955 | 0.027 |
| *Clostridium_innocuum_group* | Inverse variance weighted | 9 | -0.146 | 0.085 | 0.864 | 0.731-1.021 | 0.085 |
|  | MR Egger | 9 | 0.443 | 0.411 | 1.557 | 0.696-3.483 | 0.317 |
|  | Weighted median | 9 | -0.18 | 0.117 | 0.835 | 0.664-1.05 | 0.123 |
|  | Weighted mode | 9 | -0.213 | 0.2 | 0.808 | 0.546-1.197 | 0.318 |
|  | Maximum likelihood | 9 | -0.155 | 0.09 | 0.856 | 0.718-1.02 | 0.082 |
|  | cML-MA-BIC | 9 | -0.184 | 0.1 | 0.832 | 0.683-1.012 | 0.066 |
| *Clostridiumsensustricto1* | Inverse variance weighted | 7 | -0.011 | 0.156 | 0.989 | 0.729-1.342 | 0.944 |
|  | MR Egger | 7 | -0.283 | 0.411 | 0.754 | 0.337-1.687 | 0.522 |
|  | Weighted median | 7 | 0.03 | 0.203 | 1.03 | 0.692-1.534 | 0.883 |
|  | Weighted mode | 7 | 0.005 | 0.26 | 1.005 | 0.605-1.672 | 0.984 |
|  | Maximum likelihood | 7 | -0.011 | 0.158 | 0.989 | 0.725-1.348 | 0.943 |
|  | cML-MA-BIC | 7 | -0.019 | 0.16 | 0.981 | 0.716-1.343 | 0.905 |
| *Collinsella* | Inverse variance weighted | 9 | 0.069 | 0.203 | 1.071 | 0.72-1.594 | 0.734 |
|  | MR Egger | 9 | -0.177 | 0.803 | 0.838 | 0.174-4.046 | 0.832 |
|  | Weighted median | 9 | -0.018 | 0.246 | 0.982 | 0.606-1.591 | 0.941 |
|  | Weighted mode | 9 | 0.009 | 0.357 | 1.009 | 0.501-2.033 | 0.98 |
|  | Maximum likelihood | 9 | 0.073 | 0.181 | 1.076 | 0.755-1.534 | 0.686 |
|  | cML-MA-BIC | 9 | 0.007 | 0.199 | 1.007 | 0.682-1.486 | 0.974 |
| *Coprobacter* | Inverse variance weighted | 11 | 0.158 | 0.139 | 1.171 | 0.892-1.538 | 0.255 |
|  | MR Egger | 11 | 1.035 | 0.483 | 2.816 | 1.094-7.252 | 0.06 |
|  | Weighted median | 11 | 0.172 | 0.157 | 1.187 | 0.873-1.615 | 0.273 |
|  | Weighted mode | 11 | 0.21 | 0.3 | 1.234 | 0.685-2.223 | 0.5 |
|  | Maximum likelihood | 11 | 0.171 | 0.106 | 1.186 | 0.963-1.461 | 0.109 |
|  | cML-MA-BIC | 11 | 0.134 | 0.121 | 1.143 | 0.901-1.45 | 0.27 |
| *Coprococcus1* | Inverse variance weighted | 12 | 0.238 | 0.139 | 1.269 | 0.967-1.667 | 0.086 |
|  | MR Egger | 12 | 0.113 | 0.349 | 1.12 | 0.565-2.22 | 0.753 |
|  | Weighted median | 12 | 0.217 | 0.189 | 1.243 | 0.858-1.8 | 0.251 |
|  | Weighted mode | 12 | 0.15 | 0.266 | 1.162 | 0.69-1.956 | 0.584 |
|  | Maximum likelihood | 12 | 0.245 | 0.141 | 1.278 | 0.97-1.684 | 0.082 |
|  | cML-MA-BIC | 12 | 0.239 | 0.142 | 1.27 | 0.961-1.679 | 0.093 |
| *Coprococcus2* | Inverse variance weighted | 8 | 0.023 | 0.17 | 1.024 | 0.734-1.428 | 0.89 |
|  | MR Egger | 8 | 0.593 | 1.423 | 1.809 | 0.111-29.436 | 0.691 |
|  | Weighted median | 8 | 0.226 | 0.207 | 1.253 | 0.835-1.881 | 0.276 |
|  | Weighted mode | 8 | 0.299 | 0.3 | 1.349 | 0.749-2.427 | 0.352 |
|  | Maximum likelihood | 8 | 0.024 | 0.162 | 1.025 | 0.745-1.409 | 0.88 |
|  | cML-MA-BIC | 8 | 0.072 | 0.18 | 1.075 | 0.755-1.531 | 0.689 |
| *Coprococcus3* | Inverse variance weighted | 9 | -0.014 | 0.202 | 0.986 | 0.664-1.464 | 0.943 |
|  | MR Egger | 9 | -0.035 | 1.202 | 0.966 | 0.092-10.18 | 0.978 |
|  | Weighted median | 9 | -0.381 | 0.253 | 0.683 | 0.416-1.121 | 0.132 |
|  | Weighted mode | 9 | -0.477 | 0.402 | 0.621 | 0.283-1.364 | 0.27 |
|  | Maximum likelihood | 9 | -0.015 | 0.184 | 0.985 | 0.686-1.413 | 0.934 |
|  | cML-MA-BIC | 9 | -0.032 | 0.191 | 0.968 | 0.666-1.408 | 0.865 |
| *Defluviitaleaceae_UCG-011* | Inverse variance weighted | 9 | 0.272 | 0.151 | 1.312 | 0.976-1.765 | 0.072 |
|  | MR Egger | 9 | -0.433 | 0.514 | 0.649 | 0.237-1.775 | 0.427 |
|  | Weighted median | 9 | 0.404 | 0.19 | 1.497 | 1.031-2.174 | 0.034 |
|  | Weighted mode | 9 | 0.435 | 0.332 | 1.544 | 0.805-2.962 | 0.227 |
|  | Maximum likelihood | 9 | 0.292 | 0.136 | 1.34 | 1.026-1.749 | 0.032 |
|  | cML-MA-BIC | 9 | 0.278 | 0.146 | 1.321 | 0.992-1.76 | 0.057 |
| *Desulfovibrio* | Inverse variance weighted | 10 | 0.058 | 0.126 | 1.06 | 0.828-1.357 | 0.645 |
|  | MR Egger | 10 | -0.419 | 0.374 | 0.658 | 0.316-1.368 | 0.295 |
|  | Weighted median | 10 | 0.058 | 0.167 | 1.059 | 0.763-1.47 | 0.731 |
|  | Weighted mode | 10 | -0.046 | 0.245 | 0.955 | 0.591-1.545 | 0.856 |
|  | Maximum likelihood | 10 | 0.06 | 0.129 | 1.062 | 0.825-1.367 | 0.64 |
|  | cML-MA-BIC | 10 | 0.017 | 0.139 | 1.017 | 0.774-1.337 | 0.903 |
| *Dialister* | Inverse variance weighted | 11 | -0.029 | 0.136 | 0.971 | 0.745-1.267 | 0.829 |
|  | MR Egger | 11 | -0.455 | 0.557 | 0.634 | 0.213-1.888 | 0.434 |
|  | Weighted median | 11 | -0.011 | 0.176 | 0.989 | 0.701-1.397 | 0.952 |
|  | Weighted mode | 11 | -0.023 | 0.283 | 0.977 | 0.561-1.701 | 0.937 |
|  | Maximum likelihood | 11 | -0.029 | 0.137 | 0.971 | 0.743-1.271 | 0.832 |
|  | cML-MA-BIC | 11 | -0.025 | 0.139 | 0.975 | 0.743-1.279 | 0.855 |
| *Dorea* | Inverse variance weighted | 10 | 0.032 | 0.174 | 1.032 | 0.734-1.452 | 0.855 |
|  | MR Egger | 10 | 0.675 | 0.486 | 1.964 | 0.758-5.091 | 0.202 |
|  | Weighted median | 10 | -0.04 | 0.231 | 0.961 | 0.611-1.51 | 0.863 |
|  | Weighted mode | 10 | -0.093 | 0.328 | 0.912 | 0.479-1.735 | 0.784 |
|  | Maximum likelihood | 10 | 0.033 | 0.177 | 1.033 | 0.73-1.463 | 0.854 |
|  | cML-MA-BIC | 10 | 0.018 | 0.181 | 1.018 | 0.714-1.452 | 0.921 |
| *Eggerthella* | Inverse variance weighted | 10 | -0.145 | 0.098 | 0.865 | 0.713-1.049 | 0.14 |
|  | MR Egger | 10 | -0.25 | 0.481 | 0.779 | 0.303-2.001 | 0.618 |
|  | Weighted median | 10 | -0.17 | 0.134 | 0.844 | 0.648-1.098 | 0.206 |
|  | Weighted mode | 10 | -0.196 | 0.217 | 0.822 | 0.537-1.259 | 0.391 |
|  | Maximum likelihood | 10 | -0.152 | 0.099 | 0.859 | 0.708-1.043 | 0.124 |
|  | cML-MA-BIC | 10 | -0.143 | 0.102 | 0.866 | 0.71-1.058 | 0.159 |
| *Eisenbergiella* | Inverse variance weighted | 11 | -0.047 | 0.098 | 0.955 | 0.787-1.157 | 0.636 |
|  | MR Egger | 11 | -0.284 | 0.762 | 0.753 | 0.169-3.355 | 0.718 |
|  | Weighted median | 11 | -0.195 | 0.129 | 0.823 | 0.639-1.06 | 0.132 |
|  | Weighted mode | 11 | -0.247 | 0.213 | 0.781 | 0.514-1.187 | 0.274 |
|  | Maximum likelihood | 11 | -0.046 | 0.095 | 0.955 | 0.793-1.15 | 0.626 |
|  | cML-MA-BIC | 11 | -0.065 | 0.101 | 0.937 | 0.769-1.141 | 0.515 |
| *Enterorhabdus* | Inverse variance weighted | 6 | -0.123 | 0.147 | 0.884 | 0.662-1.181 | 0.404 |
|  | MR Egger | 6 | -0.407 | 0.388 | 0.665 | 0.311-1.424 | 0.353 |
|  | Weighted median | 6 | -0.184 | 0.19 | 0.832 | 0.573-1.209 | 0.335 |
|  | Weighted mode | 6 | -0.273 | 0.258 | 0.761 | 0.459-1.261 | 0.338 |
|  | Maximum likelihood | 6 | -0.124 | 0.148 | 0.883 | 0.661-1.181 | 0.402 |
|  | cML-MA-BIC | 6 | -0.117 | 0.153 | 0.89 | 0.66-1.2 | 0.444 |
| *Erysipelatoclostridium* | Inverse variance weighted | 15 | -0.046 | 0.109 | 0.955 | 0.771-1.183 | 0.674 |
|  | MR Egger | 15 | -0.005 | 0.445 | 0.995 | 0.416-2.378 | 0.991 |
|  | Weighted median | 15 | -0.146 | 0.145 | 0.864 | 0.65-1.148 | 0.313 |
|  | Weighted mode | 15 | -0.177 | 0.238 | 0.838 | 0.526-1.335 | 0.469 |
|  | Maximum likelihood | 15 | -0.046 | 0.106 | 0.955 | 0.776-1.174 | 0.661 |
|  | cML-MA-BIC | 15 | -0.082 | 0.114 | 0.921 | 0.737-1.15 | 0.468 |
| *Erysipelotrichaceae_UCG-003* | Inverse variance weighted | 16 | 0.127 | 0.117 | 1.136 | 0.903-1.43 | 0.277 |
|  | MR Egger | 16 | 0.002 | 0.321 | 1.002 | 0.534-1.88 | 0.994 |
|  | Weighted median | 16 | 0.014 | 0.154 | 1.014 | 0.75-1.373 | 0.926 |
|  | Weighted mode | 16 | -0.082 | 0.241 | 0.921 | 0.574-1.478 | 0.739 |
|  | Maximum likelihood | 16 | 0.133 | 0.119 | 1.142 | 0.905-1.441 | 0.263 |
|  | cML-MA-BIC | 16 | 0.126 | 0.121 | 1.134 | 0.896-1.436 | 0.296 |
| *Escherichia* | Inverse variance weighted | 10 | -0.075 | 0.215 | 0.928 | 0.608-1.415 | 0.727 |
|  | MR Egger | 10 | -0.331 | 0.702 | 0.718 | 0.181-2.844 | 0.65 |
|  | Weighted median | 10 | 0.012 | 0.219 | 1.013 | 0.659-1.557 | 0.955 |
|  | Weighted mode | 10 | 0.083 | 0.364 | 1.086 | 0.532-2.219 | 0.825 |
|  | Maximum likelihood | 10 | -0.079 | 0.158 | 0.924 | 0.679-1.259 | 0.618 |
|  | cML-MA-BIC | 10 | 0.039 | 0.182 | 1.039 | 0.727-1.486 | 0.832 |
| *Eubacterium_brachy_group* | Inverse variance weighted | 10 | -0.077 | 0.084 | 0.926 | 0.786-1.091 | 0.358 |
|  | MR Egger | 10 | -0.403 | 0.337 | 0.668 | 0.345-1.293 | 0.266 |
|  | Weighted median | 10 | -0.13 | 0.11 | 0.878 | 0.707-1.09 | 0.237 |
|  | Weighted mode | 10 | -0.114 | 0.189 | 0.893 | 0.617-1.292 | 0.562 |
|  | Maximum likelihood | 10 | -0.076 | 0.085 | 0.927 | 0.784-1.095 | 0.372 |
|  | cML-MA-BIC | 10 | -0.087 | 0.088 | 0.917 | 0.772-1.089 | 0.322 |
| *Eubacterium_coprostanoligenes_group* | Inverse variance weighted | 12 | -0.085 | 0.174 | 0.919 | 0.654-1.291 | 0.626 |
|  | MR Egger | 12 | -1.133 | 0.606 | 0.322 | 0.098-1.057 | 0.091 |
|  | Weighted median | 12 | -0.044 | 0.22 | 0.957 | 0.622-1.472 | 0.841 |
|  | Weighted mode | 12 | -0.203 | 0.351 | 0.817 | 0.41-1.626 | 0.576 |
|  | Maximum likelihood | 12 | -0.088 | 0.163 | 0.916 | 0.666-1.261 | 0.59 |
|  | cML-MA-BIC | 12 | -0.144 | 0.177 | 0.866 | 0.611-1.226 | 0.416 |
| *Eubacterium_eligens_group* | Inverse variance weighted | 7 | -0.274 | 0.289 | 0.761 | 0.432-1.34 | 0.343 |
|  | MR Egger | 7 | 0.512 | 1.162 | 1.668 | 0.171-16.252 | 0.678 |
|  | Weighted median | 7 | -0.226 | 0.27 | 0.797 | 0.47-1.353 | 0.402 |
|  | Weighted mode | 7 | -0.136 | 0.351 | 0.873 | 0.439-1.735 | 0.711 |
|  | Maximum likelihood | 7 | -0.302 | 0.205 | 0.739 | 0.495-1.104 | 0.14 |
|  | cML-MA-BIC | 7 | -0.202 | 0.23 | 0.817 | 0.521-1.282 | 0.379 |
| *Eubacterium_fissicatena_group* | Inverse variance weighted | 9 | 0.247 | 0.089 | 1.28 | 1.075-1.524 | 0.006 |
|  | MR Egger | 9 | -0.43 | 0.463 | 0.65 | 0.262-1.613 | 0.384 |
|  | Weighted median | 9 | 0.212 | 0.125 | 1.237 | 0.968-1.579 | 0.089 |
|  | Weighted mode | 9 | 0.211 | 0.176 | 1.235 | 0.875-1.743 | 0.264 |
|  | Maximum likelihood | 9 | 0.257 | 0.093 | 1.293 | 1.077-1.553 | 0.006 |
|  | cML-MA-BIC | 9 | 0.249 | 0.096 | 1.283 | 1.063-1.55 | 0.01 |
| *Eubacterium_hallii_group* | Inverse variance weighted | 16 | 0.054 | 0.136 | 1.055 | 0.808-1.378 | 0.695 |
|  | MR Egger | 16 | 0.389 | 0.272 | 1.476 | 0.865-2.517 | 0.175 |
|  | Weighted median | 16 | 0.066 | 0.174 | 1.068 | 0.76-1.5 | 0.706 |
|  | Weighted mode | 16 | -0.119 | 0.27 | 0.888 | 0.523-1.506 | 0.665 |
|  | Maximum likelihood | 16 | 0.057 | 0.117 | 1.059 | 0.842-1.332 | 0.627 |
|  | cML-MA-BIC | 16 | 0.106 | 0.128 | 1.112 | 0.865-1.43 | 0.407 |
| *Eubacterium_nodatum_group* | Inverse variance weighted | 11 | 0.011 | 0.073 | 1.011 | 0.877-1.167 | 0.876 |
|  | MR Egger | 11 | 0.199 | 0.324 | 1.221 | 0.646-2.305 | 0.554 |
|  | Weighted median | 11 | -0.019 | 0.096 | 0.982 | 0.813-1.185 | 0.847 |
|  | Weighted mode | 11 | -0.108 | 0.159 | 0.898 | 0.658-1.225 | 0.513 |
|  | Maximum likelihood | 11 | 0.012 | 0.074 | 1.012 | 0.875-1.17 | 0.873 |
|  | cML-MA-BIC | 11 | -0.002 | 0.079 | 0.998 | 0.855-1.165 | 0.978 |
| *Eubacterium_oxidoreducens_group* | Inverse variance weighted | 5 | -0.008 | 0.133 | 0.992 | 0.764-1.289 | 0.954 |
|  | MR Egger | 5 | 0.182 | 0.494 | 1.2 | 0.455-3.162 | 0.737 |
|  | Weighted median | 5 | 0.02 | 0.162 | 1.02 | 0.742-1.401 | 0.904 |
|  | Weighted mode | 5 | 0.064 | 0.229 | 1.067 | 0.681-1.671 | 0.793 |
|  | Maximum likelihood | 5 | -0.008 | 0.134 | 0.992 | 0.763-1.29 | 0.954 |
|  | cML-MA-BIC | 5 | -0.007 | 0.135 | 0.993 | 0.763-1.293 | 0.961 |
| *Eubacterium_rectale_group* | Inverse variance weighted | 8 | 0.195 | 0.226 | 1.215 | 0.78-1.894 | 0.39 |
|  | MR Egger | 8 | 0.082 | 0.892 | 1.086 | 0.189-6.238 | 0.929 |
|  | Weighted median | 8 | 0.304 | 0.252 | 1.356 | 0.827-2.223 | 0.227 |
|  | Weighted mode | 8 | 0.443 | 0.346 | 1.557 | 0.79-3.068 | 0.242 |
|  | Maximum likelihood | 8 | 0.207 | 0.19 | 1.23 | 0.847-1.785 | 0.276 |
|  | cML-MA-BIC | 8 | 0.297 | 0.215 | 1.346 | 0.882-2.053 | 0.168 |
| *Eubacterium_ruminantium_group* | Inverse variance weighted | 18 | 0.005 | 0.096 | 1.005 | 0.833-1.213 | 0.956 |
|  | MR Egger | 18 | -0.01 | 0.331 | 0.99 | 0.518-1.893 | 0.977 |
|  | Weighted median | 18 | 0.032 | 0.115 | 1.032 | 0.823-1.294 | 0.784 |
|  | Weighted mode | 18 | 0.047 | 0.186 | 1.048 | 0.728-1.508 | 0.804 |
|  | Maximum likelihood | 18 | 0.006 | 0.083 | 1.006 | 0.854-1.184 | 0.946 |
|  | cML-MA-BIC | 18 | 0.034 | 0.091 | 1.034 | 0.865-1.236 | 0.712 |
| *Eubacterium_ventriosum_group* | Inverse variance weighted | 15 | -0.113 | 0.13 | 0.894 | 0.693-1.152 | 0.385 |
|  | MR Egger | 15 | 0.885 | 0.578 | 2.423 | 0.78-7.523 | 0.15 |
|  | Weighted median | 15 | -0.198 | 0.182 | 0.821 | 0.574-1.173 | 0.279 |
|  | Weighted mode | 15 | -0.354 | 0.325 | 0.702 | 0.371-1.328 | 0.295 |
|  | Maximum likelihood | 15 | -0.111 | 0.131 | 0.895 | 0.692-1.158 | 0.4 |
|  | cML-MA-BIC | 15 | -0.123 | 0.134 | 0.884 | 0.68-1.15 | 0.359 |
| *Eubacterium_xylanophilum_group* | Inverse variance weighted | 9 | 0.185 | 0.182 | 1.203 | 0.842-1.72 | 0.309 |
|  | MR Egger | 9 | 0.284 | 0.582 | 1.328 | 0.425-4.156 | 0.641 |
|  | Weighted median | 9 | 0.094 | 0.195 | 1.098 | 0.749-1.61 | 0.631 |
|  | Weighted mode | 9 | 0.075 | 0.254 | 1.078 | 0.655-1.775 | 0.774 |
|  | Maximum likelihood | 9 | 0.196 | 0.149 | 1.216 | 0.909-1.628 | 0.188 |
|  | cML-MA-BIC | 9 | 0.107 | 0.168 | 1.113 | 0.802-1.547 | 0.521 |
| *Faecalibacterium* | Inverse variance weighted | 10 | -0.099 | 0.132 | 0.906 | 0.699-1.175 | 0.457 |
|  | MR Egger | 10 | -0.135 | 0.259 | 0.873 | 0.526-1.451 | 0.615 |
|  | Weighted median | 10 | -0.172 | 0.177 | 0.842 | 0.595-1.19 | 0.329 |
|  | Weighted mode | 10 | -0.19 | 0.193 | 0.827 | 0.567-1.207 | 0.351 |
|  | Maximum likelihood | 10 | -0.1 | 0.133 | 0.905 | 0.697-1.175 | 0.454 |
|  | cML-MA-BIC | 10 | -0.097 | 0.134 | 0.907 | 0.698-1.18 | 0.468 |
| *Family_XIII_AD3011_group* | Inverse variance weighted | 13 | 0.274 | 0.17 | 1.316 | 0.942-1.837 | 0.107 |
|  | MR Egger | 13 | -0.543 | 0.802 | 0.581 | 0.121-2.798 | 0.512 |
|  | Weighted median | 13 | 0.247 | 0.202 | 1.28 | 0.862-1.903 | 0.221 |
|  | Weighted mode | 13 | 0.27 | 0.317 | 1.309 | 0.704-2.437 | 0.411 |
|  | Maximum likelihood | 13 | 0.291 | 0.141 | 1.338 | 1.016-1.764 | 0.038 |
|  | cML-MA-BIC | 13 | 0.166 | 0.159 | 1.18 | 0.863-1.613 | 0.299 |
| *Family_XIII_UCG-001* | Inverse variance weighted | 8 | -0.233 | 0.158 | 0.792 | 0.582-1.079 | 0.139 |
|  | MR Egger | 8 | -0.303 | 0.485 | 0.739 | 0.286-1.911 | 0.555 |
|  | Weighted median | 8 | -0.33 | 0.2 | 0.719 | 0.486-1.063 | 0.098 |
|  | Weighted mode | 8 | -0.345 | 0.307 | 0.708 | 0.388-1.292 | 0.297 |
|  | Maximum likelihood | 8 | -0.234 | 0.161 | 0.792 | 0.577-1.086 | 0.148 |
|  | cML-MA-BIC | 8 | -0.259 | 0.168 | 0.772 | 0.555-1.073 | 0.124 |
| *Flavonifractor* | Inverse variance weighted | 5 | 0.124 | 0.186 | 1.132 | 0.787-1.629 | 0.503 |
|  | MR Egger | 5 | -0.228 | 0.741 | 0.796 | 0.186-3.4 | 0.778 |
|  | Weighted median | 5 | 0.018 | 0.236 | 1.018 | 0.641-1.618 | 0.939 |
|  | Weighted mode | 5 | -0.054 | 0.319 | 0.947 | 0.507-1.771 | 0.874 |
|  | Maximum likelihood | 5 | 0.126 | 0.188 | 1.135 | 0.786-1.639 | 0.5 |
|  | cML-MA-BIC | 5 | 0.12 | 0.191 | 1.127 | 0.776-1.638 | 0.529 |
| *Fusicatenibacter* | Inverse variance weighted | 18 | 0.227 | 0.128 | 1.254 | 0.975-1.613 | 0.077 |
|  | MR Egger | 18 | -0.064 | 0.481 | 0.938 | 0.365-2.41 | 0.896 |
|  | Weighted median | 18 | 0.348 | 0.175 | 1.416 | 1.004-1.997 | 0.047 |
|  | Weighted mode | 18 | 0.528 | 0.321 | 1.696 | 0.903-3.183 | 0.119 |
|  | Maximum likelihood | 18 | 0.233 | 0.129 | 1.262 | 0.979-1.626 | 0.072 |
|  | cML-MA-BIC | 18 | 0.237 | 0.131 | 1.267 | 0.98-1.639 | 0.071 |
| *Gordonibacter* | Inverse variance weighted | 12 | 0.028 | 0.082 | 1.029 | 0.875-1.209 | 0.732 |
|  | MR Egger | 12 | -0.237 | 0.363 | 0.789 | 0.387-1.607 | 0.528 |
|  | Weighted median | 12 | 0.037 | 0.107 | 1.037 | 0.842-1.279 | 0.732 |
|  | Weighted mode | 12 | 0.002 | 0.179 | 1.002 | 0.705-1.424 | 0.992 |
|  | Maximum likelihood | 12 | 0.03 | 0.075 | 1.031 | 0.89-1.194 | 0.686 |
|  | cML-MA-BIC | 12 | 0.012 | 0.08 | 1.012 | 0.865-1.185 | 0.88 |
| *Haemophilus* | Inverse variance weighted | 9 | 0.148 | 0.14 | 1.16 | 0.881-1.527 | 0.29 |
|  | MR Egger | 9 | 0.235 | 0.333 | 1.265 | 0.658-2.432 | 0.504 |
|  | Weighted median | 9 | 0.078 | 0.16 | 1.081 | 0.789-1.48 | 0.628 |
|  | Weighted mode | 9 | 0.044 | 0.203 | 1.045 | 0.702-1.557 | 0.834 |
|  | Maximum likelihood | 9 | 0.157 | 0.117 | 1.17 | 0.931-1.472 | 0.178 |
|  | cML-MA-BIC | 9 | 0.088 | 0.143 | 1.092 | 0.825-1.445 | 0.538 |
| *Holdemanella* | Inverse variance weighted | 11 | 0.111 | 0.097 | 1.117 | 0.924-1.351 | 0.252 |
|  | MR Egger | 11 | 0.548 | 0.277 | 1.729 | 1.006-2.975 | 0.079 |
|  | Weighted median | 11 | 0.208 | 0.136 | 1.231 | 0.944-1.606 | 0.125 |
|  | Weighted mode | 11 | 0.249 | 0.256 | 1.283 | 0.777-2.119 | 0.353 |
|  | Maximum likelihood | 11 | 0.117 | 0.099 | 1.124 | 0.925-1.365 | 0.239 |
|  | cML-MA-BIC | 11 | 0.096 | 0.108 | 1.101 | 0.892-1.36 | 0.371 |
| *Holdemania* | Inverse variance weighted | 14 | 0.041 | 0.104 | 1.042 | 0.85-1.278 | 0.691 |
|  | MR Egger | 14 | 0.57 | 0.307 | 1.768 | 0.969-3.228 | 0.088 |
|  | Weighted median | 14 | 0.086 | 0.136 | 1.09 | 0.835-1.423 | 0.526 |
|  | Weighted mode | 14 | 0.057 | 0.211 | 1.058 | 0.7-1.601 | 0.792 |
|  | Maximum likelihood | 14 | 0.043 | 0.105 | 1.044 | 0.849-1.283 | 0.684 |
|  | cML-MA-BIC | 14 | 0.048 | 0.107 | 1.049 | 0.851-1.294 | 0.653 |
| *Howardella* | Inverse variance weighted | 9 | 0.13 | 0.093 | 1.139 | 0.95-1.365 | 0.161 |
|  | MR Egger | 9 | 0.495 | 0.367 | 1.641 | 0.8-3.367 | 0.219 |
|  | Weighted median | 9 | 0.072 | 0.112 | 1.075 | 0.863-1.339 | 0.52 |
|  | Weighted mode | 9 | 0.012 | 0.147 | 1.012 | 0.758-1.351 | 0.939 |
|  | Maximum likelihood | 9 | 0.134 | 0.081 | 1.144 | 0.975-1.341 | 0.098 |
|  | cML-MA-BIC | 9 | 0.104 | 0.093 | 1.11 | 0.924-1.333 | 0.264 |
| *Hungatella* | Inverse variance weighted | 5 | -0.192 | 0.131 | 0.825 | 0.638-1.066 | 0.142 |
|  | MR Egger | 5 | -0.833 | 0.835 | 0.435 | 0.085-2.235 | 0.392 |
|  | Weighted median | 5 | -0.167 | 0.165 | 0.846 | 0.612-1.169 | 0.312 |
|  | Weighted mode | 5 | -0.113 | 0.222 | 0.893 | 0.578-1.378 | 0.636 |
|  | Maximum likelihood | 5 | -0.201 | 0.124 | 0.818 | 0.641-1.044 | 0.106 |
|  | cML-MA-BIC | 5 | -0.188 | 0.13 | 0.829 | 0.642-1.07 | 0.149 |
| *Intestinibacter* | Inverse variance weighted | 15 | -0.048 | 0.116 | 0.953 | 0.76-1.196 | 0.68 |
|  | MR Egger | 15 | -0.389 | 0.378 | 0.678 | 0.323-1.422 | 0.323 |
|  | Weighted median | 15 | -0.021 | 0.156 | 0.979 | 0.722-1.329 | 0.894 |
|  | Weighted mode | 15 | 0.022 | 0.22 | 1.022 | 0.664-1.573 | 0.922 |
|  | Maximum likelihood | 15 | -0.047 | 0.116 | 0.954 | 0.76-1.199 | 0.688 |
|  | cML-MA-BIC | 15 | -0.026 | 0.122 | 0.974 | 0.767-1.237 | 0.829 |
| *Intestinimonas* | Inverse variance weighted | 16 | 0.005 | 0.109 | 1.005 | 0.812-1.245 | 0.961 |
|  | MR Egger | 16 | -0.406 | 0.302 | 0.666 | 0.368-1.204 | 0.2 |
|  | Weighted median | 16 | -0.028 | 0.146 | 0.973 | 0.731-1.295 | 0.849 |
|  | Weighted mode | 16 | -0.081 | 0.231 | 0.922 | 0.587-1.45 | 0.731 |
|  | Maximum likelihood | 16 | 0.005 | 0.11 | 1.005 | 0.81-1.248 | 0.961 |
|  | cML-MA-BIC | 16 | 0.002 | 0.111 | 1.002 | 0.806-1.245 | 0.989 |
| *Lachnoclostridium* | Inverse variance weighted | 13 | 0.119 | 0.147 | 1.126 | 0.844-1.503 | 0.42 |
|  | MR Egger | 13 | -0.033 | 0.501 | 0.968 | 0.363-2.584 | 0.949 |
|  | Weighted median | 13 | -0.015 | 0.203 | 0.985 | 0.661-1.467 | 0.941 |
|  | Weighted mode | 13 | -0.083 | 0.311 | 0.921 | 0.501-1.693 | 0.795 |
|  | Maximum likelihood | 13 | 0.125 | 0.15 | 1.133 | 0.845-1.52 | 0.405 |
|  | cML-MA-BIC | 13 | 0.128 | 0.152 | 1.137 | 0.844-1.532 | 0.399 |
| *Lachnospira* | Inverse variance weighted | 6 | -0.198 | 0.227 | 0.82 | 0.526-1.28 | 0.383 |
|  | MR Egger | 6 | -1.573 | 1.338 | 0.208 | 0.015-2.86 | 0.305 |
|  | Weighted median | 6 | -0.238 | 0.294 | 0.789 | 0.443-1.404 | 0.42 |
|  | Weighted mode | 6 | -0.229 | 0.412 | 0.796 | 0.355-1.784 | 0.603 |
|  | Maximum likelihood | 6 | -0.202 | 0.23 | 0.817 | 0.521-1.281 | 0.378 |
|  | cML-MA-BIC | 6 | -0.195 | 0.234 | 0.823 | 0.521-1.301 | 0.404 |
| *Lachnospiraceae_FCS020_group* | Inverse variance weighted | 12 | -0.11 | 0.156 | 0.896 | 0.661-1.216 | 0.481 |
|  | MR Egger | 12 | -0.7 | 0.382 | 0.497 | 0.235-1.051 | 0.097 |
|  | Weighted median | 12 | -0.32 | 0.18 | 0.726 | 0.51-1.034 | 0.076 |
|  | Weighted mode | 12 | -0.407 | 0.25 | 0.665 | 0.407-1.087 | 0.132 |
|  | Maximum likelihood | 12 | -0.114 | 0.134 | 0.892 | 0.686-1.16 | 0.394 |
|  | cML-MA-BIC | 12 | -0.162 | 0.147 | 0.85 | 0.637-1.135 | 0.271 |
| *Lachnospiraceae_NC2004_group* | Inverse variance weighted | 9 | 0.016 | 0.113 | 1.016 | 0.814-1.268 | 0.888 |
|  | MR Egger | 9 | 0.267 | 0.482 | 1.305 | 0.508-3.358 | 0.597 |
|  | Weighted median | 9 | -0.005 | 0.152 | 0.995 | 0.738-1.341 | 0.973 |
|  | Weighted mode | 9 | -0.171 | 0.216 | 0.843 | 0.552-1.286 | 0.45 |
|  | Maximum likelihood | 9 | 0.017 | 0.11 | 1.017 | 0.819-1.262 | 0.88 |
|  | cML-MA-BIC | 9 | 0.005 | 0.114 | 1.005 | 0.804-1.256 | 0.966 |
| *Lachnospiraceae_ND3007_group* | Inverse variance weighted | 3 | 0.143 | 0.306 | 1.154 | 0.634-2.1 | 0.64 |
|  | MR Egger | 3 | 3.962 | 5.158 | 52.549 | 0.002-1292904.366 | 0.583 |
|  | Weighted median | 3 | 0.03 | 0.378 | 1.031 | 0.491-2.163 | 0.936 |
|  | Weighted mode | 3 | -0.009 | 0.421 | 0.991 | 0.434-2.262 | 0.985 |
|  | Maximum likelihood | 3 | 0.145 | 0.308 | 1.156 | 0.632-2.112 | 0.638 |
|  | cML-MA-BIC | 3 | 0.139 | 0.311 | 1.149 | 0.624-2.113 | 0.656 |
| *Lachnospiraceae_NK4A136_group* | Inverse variance weighted | 15 | -0.074 | 0.128 | 0.929 | 0.722-1.195 | 0.566 |
|  | MR Egger | 15 | 0.04 | 0.263 | 1.041 | 0.621-1.745 | 0.88 |
|  | Weighted median | 15 | -0.145 | 0.17 | 0.865 | 0.62-1.208 | 0.396 |
|  | Weighted mode | 15 | 0.069 | 0.199 | 1.071 | 0.726-1.581 | 0.734 |
|  | Maximum likelihood | 15 | -0.078 | 0.119 | 0.925 | 0.732-1.169 | 0.515 |
|  | cML-MA-BIC | 15 | -0.091 | 0.124 | 0.913 | 0.716-1.164 | 0.464 |
| *Lachnospiraceae_UCG-001* | Inverse variance weighted | 13 | -0.095 | 0.108 | 0.91 | 0.736-1.124 | 0.38 |
|  | MR Egger | 13 | 0.5 | 0.467 | 1.649 | 0.66-4.117 | 0.307 |
|  | Weighted median | 13 | 0.025 | 0.146 | 1.025 | 0.77-1.366 | 0.865 |
|  | Weighted mode | 13 | 0.136 | 0.249 | 1.145 | 0.702-1.867 | 0.596 |
|  | Maximum likelihood | 13 | -0.095 | 0.11 | 0.909 | 0.733-1.129 | 0.388 |
|  | cML-MA-BIC | 13 | -0.073 | 0.117 | 0.93 | 0.739-1.17 | 0.536 |
| *Lachnospiraceae_UCG-004* | Inverse variance weighted | 12 | -0.022 | 0.146 | 0.978 | 0.734-1.302 | 0.878 |
|  | MR Egger | 12 | -0.485 | 0.605 | 0.616 | 0.188-2.016 | 0.442 |
|  | Weighted median | 12 | 0.009 | 0.187 | 1.009 | 0.699-1.457 | 0.96 |
|  | Weighted mode | 12 | 0.044 | 0.286 | 1.045 | 0.596-1.83 | 0.881 |
|  | Maximum likelihood | 12 | -0.023 | 0.148 | 0.978 | 0.732-1.306 | 0.878 |
|  | cML-MA-BIC | 12 | -0.015 | 0.15 | 0.985 | 0.734-1.32 | 0.918 |
| *Lachnospiraceae_UCG-008* | Inverse variance weighted | 10 | 0.034 | 0.11 | 1.034 | 0.835-1.282 | 0.757 |
|  | MR Egger | 10 | 0.363 | 0.578 | 1.437 | 0.463-4.463 | 0.548 |
|  | Weighted median | 10 | 0.09 | 0.147 | 1.095 | 0.82-1.461 | 0.54 |
|  | Weighted mode | 10 | 0.233 | 0.254 | 1.262 | 0.767-2.077 | 0.383 |
|  | Maximum likelihood | 10 | 0.036 | 0.105 | 1.037 | 0.844-1.273 | 0.733 |
|  | cML-MA-BIC | 10 | 0.028 | 0.108 | 1.028 | 0.833-1.27 | 0.794 |
| *Lachnospiraceae_UCG-010* | Inverse variance weighted | 10 | 0.024 | 0.153 | 1.024 | 0.759-1.381 | 0.878 |
|  | MR Egger | 10 | -0.219 | 0.467 | 0.803 | 0.321-2.007 | 0.652 |
|  | Weighted median | 10 | -0.055 | 0.203 | 0.946 | 0.636-1.409 | 0.786 |
|  | Weighted mode | 10 | -0.063 | 0.335 | 0.939 | 0.486-1.812 | 0.855 |
|  | Maximum likelihood | 10 | 0.025 | 0.155 | 1.025 | 0.757-1.388 | 0.872 |
|  | cML-MA-BIC | 10 | 0.015 | 0.158 | 1.015 | 0.744-1.384 | 0.926 |
| *Lactobacillus* | Inverse variance weighted | 8 | -0.059 | 0.104 | 0.943 | 0.768-1.157 | 0.572 |
|  | MR Egger | 8 | 0.213 | 0.268 | 1.238 | 0.732-2.092 | 0.456 |
|  | Weighted median | 8 | 0.028 | 0.138 | 1.028 | 0.785-1.346 | 0.84 |
|  | Weighted mode | 8 | 0.045 | 0.163 | 1.046 | 0.76-1.44 | 0.791 |
|  | Maximum likelihood | 8 | -0.06 | 0.106 | 0.941 | 0.764-1.16 | 0.57 |
|  | cML-MA-BIC | 8 | -0.048 | 0.11 | 0.953 | 0.768-1.182 | 0.66 |
| *Lactococcus* | Inverse variance weighted | 9 | -0.039 | 0.089 | 0.962 | 0.808-1.146 | 0.664 |
|  | MR Egger | 9 | -0.077 | 0.4 | 0.926 | 0.423-2.027 | 0.853 |
|  | Weighted median | 9 | 0.053 | 0.114 | 1.054 | 0.843-1.319 | 0.644 |
|  | Weighted mode | 9 | 0.069 | 0.186 | 1.072 | 0.744-1.544 | 0.72 |
|  | Maximum likelihood | 9 | -0.041 | 0.091 | 0.96 | 0.804-1.147 | 0.654 |
|  | cML-MA-BIC | 9 | -0.035 | 0.092 | 0.965 | 0.805-1.157 | 0.702 |
| *Marvinbryantia* | Inverse variance weighted | 10 | -0.26 | 0.197 | 0.771 | 0.524-1.135 | 0.187 |
|  | MR Egger | 10 | -0.66 | 0.808 | 0.517 | 0.106-2.522 | 0.438 |
|  | Weighted median | 10 | -0.131 | 0.242 | 0.877 | 0.546-1.409 | 0.587 |
|  | Weighted mode | 10 | 0.29 | 0.521 | 1.336 | 0.481-3.71 | 0.592 |
|  | Maximum likelihood | 10 | -0.26 | 0.153 | 0.771 | 0.572-1.04 | 0.089 |
|  | cML-MA-BIC | 10 | -0.25 | 0.169 | 0.779 | 0.56-1.084 | 0.138 |
| *Methanobrevibacter* | Inverse variance weighted | 6 | 0.27 | 0.109 | 1.31 | 1.059-1.621 | 0.013 |
|  | MR Egger | 6 | 0.607 | 0.407 | 1.836 | 0.827-4.075 | 0.21 |
|  | Weighted median | 6 | 0.235 | 0.148 | 1.265 | 0.946-1.693 | 0.113 |
|  | Weighted mode | 6 | 0.229 | 0.165 | 1.257 | 0.91-1.737 | 0.224 |
|  | Maximum likelihood | 6 | 0.274 | 0.112 | 1.315 | 1.055-1.639 | 0.015 |
|  | cML-MA-BIC | 6 | 0.271 | 0.114 | 1.311 | 1.049-1.638 | 0.017 |
| *Odoribacter* | Inverse variance weighted | 7 | -0.351 | 0.181 | 0.704 | 0.494-1.004 | 0.053 |
|  | MR Egger | 7 | 0.232 | 0.569 | 1.261 | 0.413-3.848 | 0.701 |
|  | Weighted median | 7 | -0.344 | 0.249 | 0.709 | 0.435-1.154 | 0.166 |
|  | Weighted mode | 7 | -0.35 | 0.373 | 0.705 | 0.34-1.463 | 0.384 |
|  | Maximum likelihood | 7 | -0.36 | 0.186 | 0.698 | 0.485-1.004 | 0.053 |
|  | cML-MA-BIC | 7 | -0.35 | 0.19 | 0.705 | 0.486-1.023 | 0.065 |
| *Olsenella* | Inverse variance weighted | 11 | -0.072 | 0.074 | 0.93 | 0.805-1.076 | 0.33 |
|  | MR Egger | 11 | 0.123 | 0.246 | 1.131 | 0.699-1.83 | 0.629 |
|  | Weighted median | 11 | -0.022 | 0.096 | 0.978 | 0.81-1.182 | 0.82 |
|  | Weighted mode | 11 | 0.014 | 0.143 | 1.014 | 0.766-1.343 | 0.923 |
|  | Maximum likelihood | 11 | -0.072 | 0.075 | 0.93 | 0.804-1.077 | 0.333 |
|  | cML-MA-BIC | 11 | -0.072 | 0.075 | 0.931 | 0.803-1.079 | 0.34 |
| *Oscillibacter* | Inverse variance weighted | 14 | -0.048 | 0.12 | 0.953 | 0.754-1.206 | 0.691 |
|  | MR Egger | 14 | 0.078 | 0.469 | 1.081 | 0.431-2.711 | 0.87 |
|  | Weighted median | 14 | -0.006 | 0.143 | 0.994 | 0.752-1.315 | 0.968 |
|  | Weighted mode | 14 | 0.013 | 0.25 | 1.013 | 0.621-1.655 | 0.958 |
|  | Maximum likelihood | 14 | -0.049 | 0.103 | 0.952 | 0.778-1.165 | 0.634 |
|  | cML-MA-BIC | 14 | -0.011 | 0.113 | 0.989 | 0.792-1.235 | 0.923 |
| *Oscillospira* | Inverse variance weighted | 8 | -0.223 | 0.189 | 0.8 | 0.552-1.158 | 0.237 |
|  | MR Egger | 8 | -1.893 | 0.603 | 0.151 | 0.046-0.491 | 0.02 |
|  | Weighted median | 8 | -0.359 | 0.208 | 0.699 | 0.465-1.05 | 0.085 |
|  | Weighted mode | 8 | -0.465 | 0.328 | 0.628 | 0.33-1.194 | 0.199 |
|  | Maximum likelihood | 8 | -0.24 | 0.149 | 0.787 | 0.588-1.053 | 0.107 |
|  | cML-MA-BIC | 8 | -0.274 | 0.163 | 0.76 | 0.552-1.047 | 0.094 |
| *Oxalobacter* | Inverse variance weighted | 11 | 0.071 | 0.08 | 1.073 | 0.917-1.256 | 0.379 |
|  | MR Egger | 11 | -0.13 | 0.377 | 0.878 | 0.42-1.839 | 0.739 |
|  | Weighted median | 11 | 0.013 | 0.108 | 1.013 | 0.819-1.253 | 0.907 |
|  | Weighted mode | 11 | -0.007 | 0.163 | 0.993 | 0.721-1.367 | 0.966 |
|  | Maximum likelihood | 11 | 0.073 | 0.082 | 1.075 | 0.917-1.262 | 0.372 |
|  | cML-MA-BIC | 11 | 0.063 | 0.085 | 1.065 | 0.901-1.257 | 0.461 |
| *Parabacteroides* | Inverse variance weighted | 6 | -0.079 | 0.184 | 0.924 | 0.645-1.324 | 0.666 |
|  | MR Egger | 6 | -0.24 | 0.563 | 0.787 | 0.261-2.373 | 0.692 |
|  | Weighted median | 6 | -0.193 | 0.236 | 0.825 | 0.52-1.309 | 0.413 |
|  | Weighted mode | 6 | -0.198 | 0.289 | 0.821 | 0.466-1.445 | 0.524 |
|  | Maximum likelihood | 6 | -0.081 | 0.185 | 0.922 | 0.642-1.326 | 0.663 |
|  | cML-MA-BIC | 6 | -0.075 | 0.188 | 0.928 | 0.643-1.341 | 0.691 |
| *Paraprevotella* | Inverse variance weighted | 13 | -0.034 | 0.11 | 0.967 | 0.779-1.2 | 0.759 |
|  | MR Egger | 13 | 0.173 | 0.429 | 1.189 | 0.513-2.755 | 0.694 |
|  | Weighted median | 13 | -0.034 | 0.125 | 0.967 | 0.756-1.235 | 0.786 |
|  | Weighted mode | 13 | -0.009 | 0.195 | 0.991 | 0.677-1.45 | 0.962 |
|  | Maximum likelihood | 13 | -0.036 | 0.092 | 0.965 | 0.805-1.156 | 0.697 |
|  | cML-MA-BIC | 13 | -0.079 | 0.103 | 0.924 | 0.756-1.13 | 0.442 |
| *Parasutterella* | Inverse variance weighted | 14 | -0.07 | 0.106 | 0.932 | 0.757-1.148 | 0.509 |
|  | MR Egger | 14 | -0.165 | 0.295 | 0.848 | 0.476-1.512 | 0.587 |
|  | Weighted median | 14 | -0.136 | 0.145 | 0.873 | 0.657-1.161 | 0.35 |
|  | Weighted mode | 14 | -0.215 | 0.224 | 0.806 | 0.52-1.25 | 0.353 |
|  | Maximum likelihood | 14 | -0.07 | 0.108 | 0.933 | 0.755-1.151 | 0.517 |
|  | cML-MA-BIC | 14 | -0.078 | 0.109 | 0.925 | 0.746-1.145 | 0.473 |
| *Peptococcus* | Inverse variance weighted | 12 | 0.098 | 0.096 | 1.103 | 0.914-1.33 | 0.306 |
|  | MR Egger | 12 | 0.361 | 0.376 | 1.435 | 0.686-3 | 0.36 |
|  | Weighted median | 12 | 0.086 | 0.119 | 1.089 | 0.862-1.377 | 0.473 |
|  | Weighted mode | 12 | -0.108 | 0.235 | 0.898 | 0.566-1.423 | 0.655 |
|  | Maximum likelihood | 12 | 0.104 | 0.087 | 1.109 | 0.935-1.316 | 0.235 |
|  | cML-MA-BIC | 12 | 0.082 | 0.096 | 1.085 | 0.9-1.309 | 0.392 |
| *Phascolarctobacterium* | Inverse variance weighted | 9 | 0.094 | 0.143 | 1.099 | 0.83-1.455 | 0.511 |
|  | MR Egger | 9 | 0.454 | 0.677 | 1.574 | 0.418-5.936 | 0.524 |
|  | Weighted median | 9 | 0.171 | 0.186 | 1.186 | 0.824-1.706 | 0.358 |
|  | Weighted mode | 9 | 0.229 | 0.279 | 1.257 | 0.728-2.17 | 0.436 |
|  | Maximum likelihood | 9 | 0.097 | 0.145 | 1.102 | 0.829-1.466 | 0.505 |
|  | cML-MA-BIC | 9 | 0.092 | 0.147 | 1.097 | 0.822-1.463 | 0.531 |
| *Prevotella7* | Inverse variance weighted | 11 | 0.039 | 0.084 | 1.04 | 0.882-1.226 | 0.64 |
|  | MR Egger | 11 | 0.556 | 0.484 | 1.744 | 0.676-4.498 | 0.28 |
|  | Weighted median | 11 | -0.001 | 0.108 | 0.999 | 0.808-1.235 | 0.991 |
|  | Weighted mode | 11 | 0.015 | 0.194 | 1.016 | 0.694-1.487 | 0.939 |
|  | Maximum likelihood | 11 | 0.042 | 0.078 | 1.042 | 0.895-1.214 | 0.593 |
|  | cML-MA-BIC | 11 | 0.023 | 0.083 | 1.023 | 0.869-1.204 | 0.784 |
| *Prevotella9* | Inverse variance weighted | 15 | -0.065 | 0.118 | 0.937 | 0.743-1.181 | 0.583 |
|  | MR Egger | 15 | -0.289 | 0.351 | 0.749 | 0.377-1.491 | 0.426 |
|  | Weighted median | 15 | -0.114 | 0.143 | 0.892 | 0.674-1.181 | 0.426 |
|  | Weighted mode | 15 | -0.291 | 0.26 | 0.748 | 0.449-1.246 | 0.283 |
|  | Maximum likelihood | 15 | -0.065 | 0.101 | 0.937 | 0.769-1.141 | 0.517 |
|  | cML-MA-BIC | 15 | -0.088 | 0.105 | 0.916 | 0.745-1.126 | 0.406 |
| *Rikenellaceae_RC9_gut_group* | Inverse variance weighted | 11 | 0.06 | 0.113 | 1.062 | 0.851-1.326 | 0.593 |
|  | MR Egger | 11 | -1.148 | 0.62 | 0.317 | 0.094-1.071 | 0.097 |
|  | Weighted median | 11 | 0.007 | 0.113 | 1.007 | 0.807-1.256 | 0.951 |
|  | Weighted mode | 11 | -0.049 | 0.166 | 0.953 | 0.688-1.319 | 0.775 |
|  | Maximum likelihood | 11 | 0.069 | 0.077 | 1.071 | 0.92-1.246 | 0.375 |
|  | cML-MA-BIC | 11 | -0.039 | 0.085 | 0.962 | 0.815-1.136 | 0.649 |
| *Romboutsia* | Inverse variance weighted | 13 | -0.064 | 0.139 | 0.938 | 0.714-1.233 | 0.647 |
|  | MR Egger | 13 | -0.088 | 0.421 | 0.916 | 0.401-2.092 | 0.839 |
|  | Weighted median | 13 | -0.016 | 0.187 | 0.984 | 0.682-1.42 | 0.93 |
|  | Weighted mode | 13 | -0.051 | 0.304 | 0.95 | 0.524-1.725 | 0.87 |
|  | Maximum likelihood | 13 | -0.066 | 0.134 | 0.936 | 0.72-1.219 | 0.625 |
|  | cML-MA-BIC | 13 | -0.035 | 0.145 | 0.966 | 0.727-1.283 | 0.81 |
| *Roseburia* | Inverse variance weighted | 14 | 0.185 | 0.145 | 1.204 | 0.905-1.601 | 0.203 |
|  | MR Egger | 14 | -0.459 | 0.446 | 0.632 | 0.264-1.514 | 0.324 |
|  | Weighted median | 14 | 0.202 | 0.199 | 1.224 | 0.828-1.809 | 0.312 |
|  | Weighted mode | 14 | 0.182 | 0.279 | 1.199 | 0.694-2.074 | 0.527 |
|  | Maximum likelihood | 14 | 0.193 | 0.148 | 1.213 | 0.908-1.62 | 0.191 |
|  | cML-MA-BIC | 14 | 0.178 | 0.152 | 1.195 | 0.887-1.609 | 0.241 |
| *Ruminiclostridium5* | Inverse variance weighted | 11 | -0.096 | 0.161 | 0.909 | 0.663-1.246 | 0.553 |
|  | MR Egger | 11 | 0.912 | 0.673 | 2.489 | 0.666-9.305 | 0.208 |
|  | Weighted median | 11 | -0.162 | 0.208 | 0.85 | 0.565-1.278 | 0.435 |
|  | Weighted mode | 11 | -0.215 | 0.32 | 0.807 | 0.43-1.512 | 0.518 |
|  | Maximum likelihood | 11 | -0.098 | 0.164 | 0.907 | 0.658-1.249 | 0.549 |
|  | cML-MA-BIC | 11 | -0.122 | 0.172 | 0.885 | 0.632-1.238 | 0.476 |
| *Ruminiclostridium6* | Inverse variance weighted | 15 | 0.039 | 0.167 | 1.04 | 0.75-1.441 | 0.815 |
|  | MR Egger | 15 | -0.305 | 0.416 | 0.737 | 0.326-1.665 | 0.476 |
|  | Weighted median | 15 | 0.056 | 0.187 | 1.058 | 0.734-1.524 | 0.764 |
|  | Weighted mode | 15 | 0.027 | 0.267 | 1.027 | 0.609-1.734 | 0.921 |
|  | Maximum likelihood | 15 | 0.043 | 0.129 | 1.044 | 0.811-1.344 | 0.74 |
|  | cML-MA-BIC | 15 | 0.087 | 0.142 | 1.091 | 0.825-1.442 | 0.542 |
| *Ruminiclostridium9* | Inverse variance weighted | 9 | -0.032 | 0.186 | 0.968 | 0.673-1.394 | 0.862 |
|  | MR Egger | 9 | 0.612 | 0.844 | 1.844 | 0.353-9.637 | 0.492 |
|  | Weighted median | 9 | 0.043 | 0.24 | 1.044 | 0.652-1.672 | 0.857 |
|  | Weighted mode | 9 | 0.215 | 0.362 | 1.24 | 0.61-2.52 | 0.569 |
|  | Maximum likelihood | 9 | -0.032 | 0.187 | 0.968 | 0.671-1.398 | 0.863 |
|  | cML-MA-BIC | 9 | -0.026 | 0.19 | 0.974 | 0.672-1.413 | 0.891 |
| *Ruminococcaceae_NK4A214_group* | Inverse variance weighted | 13 | 0.057 | 0.137 | 1.059 | 0.81-1.384 | 0.676 |
|  | MR Egger | 13 | 0.037 | 0.452 | 1.038 | 0.428-2.519 | 0.935 |
|  | Weighted median | 13 | 0.147 | 0.19 | 1.159 | 0.799-1.681 | 0.438 |
|  | Weighted mode | 13 | 0.266 | 0.26 | 1.305 | 0.784-2.172 | 0.326 |
|  | Maximum likelihood | 13 | 0.059 | 0.139 | 1.061 | 0.808-1.394 | 0.67 |
|  | cML-MA-BIC | 13 | 0.101 | 0.151 | 1.106 | 0.822-1.488 | 0.506 |
| *Ruminococcaceae_UCG-002* | Inverse variance weighted | 22 | -0.233 | 0.107 | 0.792 | 0.643-0.977 | 0.029 |
|  | MR Egger | 22 | -0.316 | 0.287 | 0.729 | 0.416-1.28 | 0.284 |
|  | Weighted median | 22 | -0.167 | 0.152 | 0.846 | 0.628-1.139 | 0.271 |
|  | Weighted mode | 22 | -0.142 | 0.243 | 0.867 | 0.539-1.397 | 0.565 |
|  | Maximum likelihood | 22 | -0.231 | 0.105 | 0.794 | 0.646-0.975 | 0.028 |
|  | cML-MA-BIC | 22 | -0.18 | 0.111 | 0.835 | 0.672-1.039 | 0.106 |
| *Ruminococcaceae_UCG-003* | Inverse variance weighted | 12 | 0.113 | 0.132 | 1.12 | 0.864-1.452 | 0.392 |
|  | MR Egger | 12 | -0.47 | 0.433 | 0.625 | 0.267-1.461 | 0.303 |
|  | Weighted median | 12 | 0.028 | 0.177 | 1.029 | 0.727-1.456 | 0.873 |
|  | Weighted mode | 12 | -0.079 | 0.25 | 0.924 | 0.566-1.51 | 0.76 |
|  | Maximum likelihood | 12 | 0.117 | 0.134 | 1.124 | 0.864-1.463 | 0.383 |
|  | cML-MA-BIC | 12 | 0.1 | 0.139 | 1.105 | 0.841-1.452 | 0.474 |
| *Ruminococcaceae_UCG-004* | Inverse variance weighted | 11 | -0.058 | 0.131 | 0.944 | 0.73-1.221 | 0.658 |
|  | MR Egger | 11 | -0.04 | 0.768 | 0.961 | 0.213-4.325 | 0.959 |
|  | Weighted median | 11 | -0.259 | 0.178 | 0.772 | 0.545-1.095 | 0.146 |
|  | Weighted mode | 11 | -0.356 | 0.324 | 0.7 | 0.371-1.322 | 0.298 |
|  | Maximum likelihood | 11 | -0.061 | 0.123 | 0.941 | 0.739-1.199 | 0.624 |
|  | cML-MA-BIC | 11 | -0.078 | 0.13 | 0.925 | 0.716-1.194 | 0.548 |
| *Ruminococcaceae_UCG-005* | Inverse variance weighted | 14 | 0.189 | 0.126 | 1.208 | 0.943-1.548 | 0.134 |
|  | MR Egger | 14 | -0.445 | 0.335 | 0.641 | 0.332-1.234 | 0.208 |
|  | Weighted median | 14 | 0.24 | 0.171 | 1.272 | 0.91-1.777 | 0.159 |
|  | Weighted mode | 14 | 0.241 | 0.275 | 1.272 | 0.742-2.18 | 0.397 |
|  | Maximum likelihood | 14 | 0.2 | 0.128 | 1.222 | 0.951-1.569 | 0.117 |
|  | cML-MA-BIC | 14 | 0.184 | 0.132 | 1.202 | 0.928-1.557 | 0.163 |
| *Ruminococcaceae_UCG-009* | Inverse variance weighted | 12 | 0.073 | 0.102 | 1.075 | 0.881-1.313 | 0.475 |
|  | MR Egger | 12 | 0.046 | 0.427 | 1.047 | 0.453-2.42 | 0.916 |
|  | Weighted median | 12 | 0.019 | 0.14 | 1.019 | 0.774-1.342 | 0.891 |
|  | Weighted mode | 12 | -0.018 | 0.206 | 0.983 | 0.657-1.47 | 0.933 |
|  | Maximum likelihood | 12 | 0.077 | 0.102 | 1.08 | 0.883-1.32 | 0.453 |
|  | cML-MA-BIC | 12 | 0.061 | 0.108 | 1.063 | 0.86-1.312 | 0.573 |
| *Ruminococcaceae_UCG-010* | Inverse variance weighted | 6 | -0.113 | 0.173 | 0.893 | 0.636-1.255 | 0.515 |
|  | MR Egger | 6 | -0.38 | 0.475 | 0.684 | 0.27-1.734 | 0.468 |
|  | Weighted median | 6 | -0.208 | 0.213 | 0.812 | 0.535-1.233 | 0.329 |
|  | Weighted mode | 6 | -0.234 | 0.289 | 0.791 | 0.449-1.395 | 0.455 |
|  | Maximum likelihood | 6 | -0.114 | 0.175 | 0.892 | 0.634-1.256 | 0.514 |
|  | cML-MA-BIC | 6 | -0.121 | 0.177 | 0.886 | 0.626-1.253 | 0.494 |
| *Ruminococcaceae_UCG-011* | Inverse variance weighted | 8 | -0.158 | 0.094 | 0.854 | 0.711-1.026 | 0.092 |
|  | MR Egger | 8 | 0.599 | 0.419 | 1.821 | 0.8-4.142 | 0.203 |
|  | Weighted median | 8 | -0.071 | 0.117 | 0.931 | 0.74-1.172 | 0.544 |
|  | Weighted mode | 8 | -0.05 | 0.155 | 0.952 | 0.702-1.29 | 0.759 |
|  | Maximum likelihood | 8 | -0.163 | 0.087 | 0.849 | 0.717-1.006 | 0.059 |
|  | cML-MA-BIC | 8 | -0.147 | 0.093 | 0.863 | 0.719-1.037 | 0.115 |
| *Ruminococcaceae_UCG-013* | Inverse variance weighted | 12 | -0.004 | 0.169 | 0.996 | 0.715-1.386 | 0.98 |
|  | MR Egger | 12 | 0.308 | 0.491 | 1.361 | 0.52-3.566 | 0.544 |
|  | Weighted median | 12 | -0.155 | 0.199 | 0.856 | 0.58-1.264 | 0.434 |
|  | Weighted mode | 12 | -0.238 | 0.351 | 0.788 | 0.396-1.569 | 0.512 |
|  | Maximum likelihood | 12 | -0.005 | 0.149 | 0.995 | 0.744-1.332 | 0.975 |
|  | cML-MA-BIC | 12 | -0.033 | 0.157 | 0.968 | 0.712-1.316 | 0.834 |
| *Ruminococcaceae_UCG-014* | Inverse variance weighted | 11 | -0.134 | 0.131 | 0.875 | 0.676-1.131 | 0.308 |
|  | MR Egger | 11 | -0.345 | 0.311 | 0.708 | 0.385-1.304 | 0.297 |
|  | Weighted median | 11 | -0.196 | 0.178 | 0.822 | 0.58-1.166 | 0.271 |
|  | Weighted mode | 11 | -0.198 | 0.216 | 0.821 | 0.538-1.253 | 0.381 |
|  | Maximum likelihood | 11 | -0.136 | 0.132 | 0.873 | 0.674-1.132 | 0.306 |
|  | cML-MA-BIC | 11 | -0.133 | 0.133 | 0.876 | 0.675-1.137 | 0.32 |
| *Ruminococcus1* | Inverse variance weighted | 10 | 0.042 | 0.149 | 1.043 | 0.779-1.397 | 0.779 |
|  | MR Egger | 10 | -0.643 | 0.397 | 0.526 | 0.241-1.145 | 0.144 |
|  | Weighted median | 10 | -0.013 | 0.194 | 0.987 | 0.674-1.443 | 0.945 |
|  | Weighted mode | 10 | -0.121 | 0.283 | 0.886 | 0.509-1.542 | 0.678 |
|  | Maximum likelihood | 10 | 0.043 | 0.152 | 1.044 | 0.775-1.407 | 0.776 |
|  | cML-MA-BIC | 10 | 0.021 | 0.158 | 1.022 | 0.749-1.393 | 0.893 |
| *Ruminococcus2* | Inverse variance weighted | 15 | 0.114 | 0.114 | 1.121 | 0.896-1.402 | 0.318 |
|  | MR Egger | 15 | -0.168 | 0.275 | 0.845 | 0.493-1.45 | 0.552 |
|  | Weighted median | 15 | 0.012 | 0.15 | 1.012 | 0.753-1.359 | 0.939 |
|  | Weighted mode | 15 | 0.035 | 0.193 | 1.036 | 0.71-1.511 | 0.858 |
|  | Maximum likelihood | 15 | 0.119 | 0.116 | 1.126 | 0.897-1.414 | 0.306 |
|  | cML-MA-BIC | 15 | 0.107 | 0.119 | 1.113 | 0.882-1.404 | 0.369 |
| *Ruminococcus_gauvreauii_group* | Inverse variance weighted | 12 | -0.009 | 0.15 | 0.991 | 0.739-1.329 | 0.952 |
|  | MR Egger | 12 | -0.461 | 0.633 | 0.63 | 0.182-2.182 | 0.483 |
|  | Weighted median | 12 | -0.003 | 0.195 | 0.997 | 0.68-1.46 | 0.987 |
|  | Weighted mode | 12 | 0.042 | 0.297 | 1.043 | 0.583-1.868 | 0.889 |
|  | Maximum likelihood | 12 | -0.009 | 0.137 | 0.991 | 0.758-1.296 | 0.947 |
|  | cML-MA-BIC | 12 | -0.039 | 0.146 | 0.962 | 0.723-1.28 | 0.79 |
| *Ruminococcus_gnavus_group* | Inverse variance weighted | 12 | -0.026 | 0.15 | 0.974 | 0.727-1.306 | 0.862 |
|  | MR Egger | 12 | 0.615 | 0.709 | 1.85 | 0.461-7.422 | 0.406 |
|  | Weighted median | 12 | -0.037 | 0.147 | 0.964 | 0.723-1.285 | 0.802 |
|  | Weighted mode | 12 | -0.118 | 0.252 | 0.889 | 0.543-1.456 | 0.649 |
|  | Maximum likelihood | 12 | -0.029 | 0.1 | 0.972 | 0.799-1.182 | 0.775 |
|  | cML-MA-BIC | 12 | 0.067 | 0.111 | 1.069 | 0.859-1.329 | 0.549 |
| *Ruminococcus_torques_group* | Inverse variance weighted | 9 | -0.106 | 0.214 | 0.9 | 0.591-1.37 | 0.622 |
|  | MR Egger | 9 | 0.31 | 0.72 | 1.363 | 0.332-5.593 | 0.68 |
|  | Weighted median | 9 | -0.321 | 0.256 | 0.725 | 0.439-1.198 | 0.209 |
|  | Weighted mode | 9 | -0.465 | 0.371 | 0.628 | 0.303-1.301 | 0.246 |
|  | Maximum likelihood | 9 | -0.11 | 0.195 | 0.896 | 0.611-1.314 | 0.573 |
|  | cML-MA-BIC | 9 | -0.19 | 0.219 | 0.827 | 0.539-1.269 | 0.385 |
| *Sellimonas* | Inverse variance weighted | 9 | -0.125 | 0.074 | 0.882 | 0.763-1.02 | 0.091 |
|  | MR Egger | 9 | 0.721 | 0.432 | 2.056 | 0.882-4.791 | 0.139 |
|  | Weighted median | 9 | -0.193 | 0.102 | 0.824 | 0.675-1.006 | 0.058 |
|  | Weighted mode | 9 | -0.213 | 0.153 | 0.808 | 0.599-1.09 | 0.201 |
|  | Maximum likelihood | 9 | -0.128 | 0.076 | 0.88 | 0.759-1.021 | 0.092 |
|  | cML-MA-BIC | 9 | -0.138 | 0.079 | 0.871 | 0.747-1.017 | 0.08 |
| *Senegalimassilia* | Inverse variance weighted | 5 | 0.019 | 0.215 | 1.019 | 0.669-1.553 | 0.929 |
|  | MR Egger | 5 | 0.488 | 0.91 | 1.629 | 0.274-9.688 | 0.629 |
|  | Weighted median | 5 | 0 | 0.226 | 1 | 0.642-1.558 | 0.999 |
|  | Weighted mode | 5 | 0.135 | 0.389 | 1.145 | 0.534-2.454 | 0.746 |
|  | Maximum likelihood | 5 | 0.021 | 0.171 | 1.021 | 0.73-1.428 | 0.904 |
|  | cML-MA-BIC | 5 | 0.065 | 0.193 | 1.067 | 0.731-1.559 | 0.736 |
| *Slackia* | Inverse variance weighted | 6 | 0.157 | 0.137 | 1.17 | 0.895-1.53 | 0.251 |
|  | MR Egger | 6 | 0.1 | 0.89 | 1.105 | 0.193-6.326 | 0.916 |
|  | Weighted median | 6 | 0.107 | 0.177 | 1.113 | 0.787-1.573 | 0.544 |
|  | Weighted mode | 6 | 0.109 | 0.247 | 1.116 | 0.688-1.809 | 0.676 |
|  | Maximum likelihood | 6 | 0.162 | 0.139 | 1.176 | 0.895-1.545 | 0.245 |
|  | cML-MA-BIC | 6 | 0.148 | 0.145 | 1.16 | 0.872-1.541 | 0.308 |
| *Streptococcus* | Inverse variance weighted | 13 | -0.01 | 0.168 | 0.99 | 0.712-1.377 | 0.952 |
|  | MR Egger | 13 | -0.236 | 0.605 | 0.79 | 0.242-2.585 | 0.704 |
|  | Weighted median | 13 | 0.015 | 0.214 | 1.015 | 0.667-1.545 | 0.945 |
|  | Weighted mode | 13 | 0.031 | 0.285 | 1.031 | 0.59-1.801 | 0.916 |
|  | Maximum likelihood | 13 | -0.011 | 0.15 | 0.989 | 0.737-1.328 | 0.944 |
|  | cML-MA-BIC | 13 | -0.094 | 0.161 | 0.91 | 0.664-1.248 | 0.559 |
| *Subdoligranulum* | Inverse variance weighted | 11 | -0.115 | 0.148 | 0.891 | 0.667-1.19 | 0.434 |
|  | MR Egger | 11 | -0.057 | 0.379 | 0.944 | 0.449-1.985 | 0.883 |
|  | Weighted median | 11 | -0.052 | 0.193 | 0.949 | 0.651-1.385 | 0.787 |
|  | Weighted mode | 11 | 0.059 | 0.312 | 1.061 | 0.576-1.956 | 0.853 |
|  | Maximum likelihood | 11 | -0.118 | 0.151 | 0.889 | 0.661-1.194 | 0.433 |
|  | cML-MA-BIC | 11 | -0.114 | 0.153 | 0.892 | 0.662-1.203 | 0.454 |
| *Sutterella* | Inverse variance weighted | 12 | 0.196 | 0.135 | 1.217 | 0.934-1.585 | 0.146 |
|  | MR Egger | 12 | 0.563 | 0.583 | 1.755 | 0.56-5.506 | 0.358 |
|  | Weighted median | 12 | 0.286 | 0.176 | 1.331 | 0.942-1.881 | 0.105 |
|  | Weighted mode | 12 | 0.355 | 0.262 | 1.426 | 0.852-2.385 | 0.204 |
|  | Maximum likelihood | 12 | 0.199 | 0.136 | 1.22 | 0.934-1.595 | 0.144 |
|  | cML-MA-BIC | 12 | 0.203 | 0.138 | 1.225 | 0.936-1.605 | 0.14 |
| *Terrisporobacter* | Inverse variance weighted | 5 | 0.012 | 0.166 | 1.012 | 0.731-1.402 | 0.943 |
|  | MR Egger | 5 | -0.625 | 0.469 | 0.535 | 0.213-1.343 | 0.275 |
|  | Weighted median | 5 | -0.013 | 0.208 | 0.988 | 0.657-1.484 | 0.952 |
|  | Weighted mode | 5 | -0.003 | 0.295 | 0.997 | 0.56-1.777 | 0.993 |
|  | Maximum likelihood | 5 | 0.012 | 0.159 | 1.012 | 0.741-1.384 | 0.938 |
|  | cML-MA-BIC | 5 | -0.002 | 0.166 | 0.998 | 0.72-1.382 | 0.989 |
| *Turicibacter* | Inverse variance weighted | 10 | -0.114 | 0.121 | 0.892 | 0.703-1.132 | 0.346 |
|  | MR Egger | 10 | -0.156 | 0.551 | 0.856 | 0.291-2.518 | 0.784 |
|  | Weighted median | 10 | -0.159 | 0.159 | 0.853 | 0.625-1.165 | 0.318 |
|  | Weighted mode | 10 | -0.192 | 0.256 | 0.825 | 0.5-1.362 | 0.472 |
|  | Maximum likelihood | 10 | -0.115 | 0.122 | 0.892 | 0.702-1.133 | 0.349 |
|  | cML-MA-BIC | 10 | -0.167 | 0.136 | 0.846 | 0.648-1.103 | 0.217 |
| *Tyzzerella3* | Inverse variance weighted | 13 | 0.034 | 0.084 | 1.035 | 0.877-1.22 | 0.686 |
|  | MR Egger | 13 | 0.046 | 0.505 | 1.047 | 0.389-2.817 | 0.93 |
|  | Weighted median | 13 | -0.077 | 0.12 | 0.926 | 0.732-1.173 | 0.524 |
|  | Weighted mode | 13 | -0.121 | 0.16 | 0.886 | 0.647-1.212 | 0.464 |
|  | Maximum likelihood | 13 | 0.036 | 0.085 | 1.037 | 0.877-1.226 | 0.673 |
|  | cML-MA-BIC | 13 | 0.019 | 0.09 | 1.019 | 0.854-1.216 | 0.832 |
| *Veillonella* | Inverse variance weighted | 6 | 0.281 | 0.163 | 1.325 | 0.962-1.824 | 0.085 |
|  | MR Egger | 6 | -0.093 | 1.3 | 0.911 | 0.071-11.64 | 0.946 |
|  | Weighted median | 6 | 0.325 | 0.197 | 1.384 | 0.94-2.037 | 0.099 |
|  | Weighted mode | 6 | 0.484 | 0.324 | 1.622 | 0.859-3.063 | 0.196 |
|  | Maximum likelihood | 6 | 0.289 | 0.167 | 1.335 | 0.962-1.853 | 0.084 |
|  | cML-MA-BIC | 6 | 0.297 | 0.171 | 1.346 | 0.963-1.881 | 0.082 |
| *Victivallis* | Inverse variance weighted | 10 | -0.032 | 0.078 | 0.969 | 0.832-1.129 | 0.685 |
|  | MR Egger | 10 | 0.54 | 0.596 | 1.716 | 0.533-5.521 | 0.392 |
|  | Weighted median | 10 | -0.06 | 0.106 | 0.942 | 0.764-1.16 | 0.571 |
|  | Weighted mode | 10 | -0.048 | 0.184 | 0.953 | 0.664-1.367 | 0.798 |
|  | Maximum likelihood | 10 | -0.033 | 0.079 | 0.968 | 0.828-1.131 | 0.679 |
|  | cML-MA-BIC | 10 | -0.027 | 0.081 | 0.973 | 0.829-1.142 | 0.738 |
| MR, Mendelian randomization; PsA, Psoriatic arthritis; SNP, single nucleotide polymorphism; SE, standard error; OR, odds ratio; CI, confidence interval; IVW, inverse variance weighted; ML, maximum likelihood. | | | | | | | |

**Supplemental Table S2:** SNPs used as IVs from gut microbiome and PsA.

| **Table S2 SNPs used as IVs from gut microbiome and PsA.** | | | | | | | | | |
| --- | --- | --- | --- | --- | --- | --- | --- | --- | --- |
| **Bacterial traits** | **SNP** | **Effect allele** | **Other allele** | **Exposure (Bacteria)** | | | **Outcome (PsA)** | | |
|  |  |  |  | **Beta** | **SE** | ***P*-value** | **Beta** | **SE** | ***P*-value** |
| *Blautia* | rs11149971 | C | T | 0.117605 | 0.02339 | 1.04E-06 | 0.106243 | 0.074521 | 0.059574 |
|  | rs113271346 | C | T | 0.078268 | 0.017212 | 6.85E-06 | -0.006078 | 0.900915 | 0.04882 |
|  | rs115043014 | A | G | 0.206605 | 0.043992 | 5.19E-06 | 0.141421 | 0.179859 | 0.105445 |
|  | rs117001700 | T | C | 0.196414 | 0.044112 | 8.84E-06 | -0.009444 | 0.934579 | 0.115053 |
|  | rs12453000 | C | T | 0.062533 | 0.012999 | 1.26E-06 | 0.025373 | 0.520183 | 0.039456 |
|  | rs16892041 | C | T | 0.06226 | 0.01418 | 8.82E-06 | 0.018446 | 0.578284 | 0.033183 |
|  | rs2788271 | G | T | 0.05756 | 0.013349 | 7.16E-06 | 0.035979 | 0.316952 | 0.035952 |
|  | rs3005511 | A | G | 0.050108 | 0.011077 | 6.19E-06 | 0.024818 | 0.400582 | 0.029525 |
|  | rs4926264 | T | C | 0.082621 | 0.017824 | 5.10E-06 | 0.018049 | 0.681985 | 0.044048 |
|  | rs67794373 | C | T | 0.060171 | 0.012344 | 1.00E-06 | 0.044735 | 0.167904 | 0.032441 |
|  | rs682885 | G | A | 0.049338 | 0.010736 | 4.49E-06 | 0.021385 | 0.460828 | 0.028998 |
|  | rs72973581 | A | G | 0.125169 | 0.026541 | 1.74E-06 | -0.032508 | 0.578502 | 0.058513 |
|  | rs7860714 | G | A | 0.050218 | 0.010985 | 4.09E-06 | -0.019616 | 0.488857 | 0.028341 |
| *Butyricicoccus* | rs10084203 | A | G | 0.05497 | 0.012356 | 8.59E-06 | -0.014204 | 0.040335 | 0.724726 |
|  | rs12034718 | A | G | 0.07012 | 0.015821 | 9.58E-06 | -0.050532 | 0.032609 | 0.121229 |
|  | rs12585793 | C | T | 0.262206 | 0.056473 | 5.79E-06 | 0.008003 | 0.082078 | 0.922327 |
|  | rs2017189 | T | G | 0.050696 | 0.011024 | 3.87E-06 | -0.049299 | 0.027125 | 0.069143 |
|  | rs4962426 | G | T | 0.061422 | 0.013598 | 7.38E-06 | -0.117549 | 0.034853 | 0.000744 |
|  | rs56221232 | T | C | 0.082803 | 0.01674 | 7.62E-07 | -0.006235 | 0.0452 | 0.890289 |
|  | rs62478070 | T | G | 0.224039 | 0.049496 | 5.94E-06 | -0.001254 | 0.086351 | 0.988415 |
|  | rs7322368 | T | C | 0.081573 | 0.018317 | 5.52E-06 | -0.028699 | 0.047356 | 0.544498 |
| *Christensenellaceae_R-7_group* | rs10461257 | G | A | 0.055197 | 0.012207 | 6.51E-06 | -0.027232 | 0.028782 | 0.344088 |
|  | rs17081797 | G | A | 0.090432 | 0.020425 | 3.34E-06 | -0.043449 | 0.055452 | 0.433315 |
|  | rs60954665 | T | G | 0.049815 | 0.011079 | 7.13E-06 | -0.028141 | 0.027119 | 0.299426 |
|  | rs62132810 | G | A | 0.082885 | 0.017962 | 5.67E-06 | 0.131844 | 0.040072 | 0.001001 |
|  | rs62190261 | A | C | 0.095839 | 0.021472 | 8.74E-06 | -0.015755 | 0.048495 | 0.745271 |
|  | rs62467127 | C | T | 0.114108 | 0.025198 | 3.25E-06 | -0.070792 | 0.086575 | 0.413534 |
|  | rs73952017 | T | C | 0.086216 | 0.019435 | 8.46E-06 | -0.060717 | 0.044428 | 0.171735 |
|  | rs78521377 | C | T | 0.124992 | 0.02748 | 5.61E-06 | 0.012893 | 0.078338 | 0.869273 |
|  | rs79150079 | C | A | 0.121547 | 0.027098 | 9.42E-06 | -0.061349 | 0.052353 | 0.241263 |
|  | rs892686 | A | G | 0.05141 | 0.011136 | 3.97E-06 | -0.009193 | 0.027251 | 0.735855 |
| *Defluviitaleaceae_UCG-011* | rs112893842 | T | C | 0.11381 | 0.023281 | 1.45E-06 | 0.053289 | 0.047542 | 0.262338 |
|  | rs1582238 | T | C | 0.080504 | 0.016726 | 1.57E-06 | 0.017463 | 0.028411 | 0.538771 |
|  | rs2892880 | G | A | 0.081776 | 0.018169 | 6.83E-06 | 0.03771 | 0.031267 | 0.227791 |
|  | rs4344384 | G | T | 0.071598 | 0.015631 | 4.83E-06 | 0.040403 | 0.027226 | 0.137815 |
|  | rs4677103 | A | G | 0.097791 | 0.019726 | 9.60E-07 | 0.091273 | 0.035889 | 0.010984 |
|  | rs55658617 | T | C | 0.174369 | 0.036223 | 2.15E-06 | 0.084809 | 0.07342 | 0.24804 |
|  | rs72731813 | T | C | 0.147383 | 0.02938 | 4.33E-07 | -0.030794 | 0.063453 | 0.627463 |
|  | rs9608282 | T | G | 0.142939 | 0.029979 | 2.52E-06 | 0.02888 | 0.076647 | 0.706331 |
|  | rs9725395 | G | A | 0.13834 | 0.029554 | 3.52E-06 | -0.059255 | 0.04289 | 0.167108 |
| *Eubacterium_fissicatena_group* | rs10147907 | T | G | 0.172263 | 0.039601 | 8.27E-06 | 0.018985 | 0.05194 | 0.71472 |
|  | rs11818408 | G | A | 0.10585 | 0.023711 | 8.20E-06 | 0.039351 | 0.027848 | 0.157635 |
|  | rs11876297 | T | C | 0.131469 | 0.028171 | 2.67E-06 | 0.028018 | 0.030664 | 0.360874 |
|  | rs151257695 | A | G | 0.20951 | 0.045485 | 3.10E-06 | -0.042181 | 0.052833 | 0.424656 |
|  | rs1768152 | T | C | 0.139489 | 0.031619 | 8.70E-06 | 0.007882 | 0.044294 | 0.858772 |
|  | rs2733072 | G | A | 0.109644 | 0.022831 | 1.49E-06 | 0.028114 | 0.027238 | 0.301998 |
|  | rs3771393 | C | T | 0.130842 | 0.026667 | 7.38E-07 | 0.088034 | 0.033603 | 0.008798 |
|  | rs6934739 | A | G | 0.111463 | 0.025279 | 9.75E-06 | 0.021673 | 0.028806 | 0.451834 |
|  | rs7104872 | G | A | 0.138612 | 0.029191 | 2.73E-06 | 0.080439 | 0.043357 | 0.063557 |
| *Family_XIII_AD3011_group* | rs11126423 | C | T | 0.09044 | 0.01963 | 5.91E-06 | -0.063319 | 0.047692 | 0.184282 |
|  | rs11736617 | A | G | 0.075949 | 0.01721 | 9.02E-06 | -0.043799 | 0.059969 | 0.465172 |
|  | rs12812672 | C | T | 0.096065 | 0.020828 | 2.56E-06 | 0.014262 | 0.052434 | 0.785628 |
|  | rs149302 | C | T | 0.064564 | 0.014322 | 7.48E-06 | -0.002196 | 0.032104 | 0.945462 |
|  | rs16840310 | G | A | 0.060805 | 0.012215 | 6.75E-07 | 0.021375 | 0.027554 | 0.437909 |
|  | rs16940167 | C | T | 0.073255 | 0.01599 | 3.91E-06 | 0.063002 | 0.034535 | 0.06811 |
|  | rs17156849 | A | G | 0.112891 | 0.024529 | 4.19E-06 | 0.044305 | 0.057519 | 0.441148 |
|  | rs62029761 | A | G | 0.128753 | 0.0276 | 3.89E-06 | -0.028154 | 0.0596 | 0.636658 |
|  | rs62200412 | T | C | 0.080085 | 0.016383 | 5.80E-07 | 0.018762 | 0.031225 | 0.547932 |
|  | rs72730932 | A | C | 0.089956 | 0.017711 | 6.89E-07 | 0.060484 | 0.046712 | 0.19538 |
|  | rs739451 | C | T | 0.064959 | 0.014753 | 7.88E-06 | 0.03382 | 0.03351 | 0.312853 |
|  | rs9276029 | G | A | 0.081138 | 0.018567 | 8.93E-06 | 0.12114 | 0.034784 | 0.000497 |
|  | rs9837139 | A | G | 0.10752 | 0.024048 | 8.71E-06 | -0.046044 | 0.04888 | 0.34621 |
| *Fusicatenibacter* | rs10439674 | G | A | 0.057213 | 0.013001 | 7.68E-06 | 0.038128 | 0.033679 | 0.257598 |
|  | rs167879 | T | C | 0.065954 | 0.014876 | 5.87E-06 | 0.008375 | 0.038134 | 0.826174 |
|  | rs1864685 | C | A | 0.04948 | 0.01081 | 4.96E-06 | 0.026076 | 0.027568 | 0.344211 |
|  | rs2025938 | A | G | 0.09673 | 0.020543 | 2.99E-06 | -0.018758 | 0.054748 | 0.731889 |
|  | rs206581 | G | A | 0.056834 | 0.012789 | 8.96E-06 | 0.022343 | 0.032931 | 0.497468 |
|  | rs2132128 | A | G | 0.077191 | 0.016034 | 1.08E-06 | -0.051814 | 0.045218 | 0.251847 |
|  | rs3303 | C | T | 0.095366 | 0.020407 | 3.94E-06 | -0.025221 | 0.055505 | 0.649542 |
|  | rs4378146 | C | A | 0.061669 | 0.012526 | 7.20E-07 | 0.030837 | 0.031231 | 0.323454 |
|  | rs60254196 | G | A | 0.049243 | 0.010936 | 5.47E-06 | 0.030765 | 0.027269 | 0.259247 |
|  | rs62187631 | C | T | 0.071057 | 0.015924 | 4.55E-06 | 0.010898 | 0.03448 | 0.751941 |
|  | rs62353480 | G | A | 0.07014 | 0.014559 | 1.57E-06 | 0.038901 | 0.036232 | 0.282975 |
|  | rs6515626 | G | A | 0.141575 | 0.031356 | 7.29E-06 | 0.09448 | 0.054801 | 0.084697 |
|  | rs704418 | T | C | 0.073912 | 0.015107 | 7.77E-07 | 0.043149 | 0.041056 | 0.293263 |
|  | rs73103914 | G | A | 0.059732 | 0.013446 | 8.30E-06 | -0.021142 | 0.037993 | 0.577896 |
|  | rs792108 | C | T | 0.050835 | 0.011384 | 8.50E-06 | 0.0173 | 0.027573 | 0.530377 |
|  | rs8028026 | G | A | 0.079215 | 0.01805 | 8.06E-06 | -0.017324 | 0.046691 | 0.710618 |
|  | rs8063430 | C | T | 0.104026 | 0.022217 | 4.93E-06 | -0.030051 | 0.06203 | 0.62806 |
|  | rs9905659 | A | G | 0.061617 | 0.013658 | 7.31E-06 | 0.000646 | 0.03522 | 0.985362 |
| *Methanobrevibacter* | rs10202904 | G | T | 0.112811 | 0.023911 | 3.09E-06 | 0.02238 | 0.027647 | 0.418248 |
|  | rs1334944 | T | C | 0.115197 | 0.025549 | 7.61E-06 | 0.007367 | 0.030272 | 0.807715 |
|  | rs4802933 | G | A | 0.135628 | 0.030814 | 9.74E-06 | 0.040995 | 0.032265 | 0.203875 |
|  | rs6776814 | C | T | 0.188956 | 0.041991 | 8.05E-06 | 0.093773 | 0.096485 | 0.331106 |
|  | rs76029318 | T | C | 0.222849 | 0.045432 | 1.08E-06 | 0.050623 | 0.056254 | 0.368176 |
|  | rs894996 | C | A | 0.214213 | 0.045604 | 3.82E-06 | 0.102273 | 0.052792 | 0.052711 |
| *Oscillospira* | rs12206468 | A | G | 0.133016 | 0.026973 | 1.04E-06 | -0.046776 | 0.050848 | 0.357619 |
|  | rs12925026 | T | C | 0.13559 | 0.030678 | 9.31E-06 | -0.121075 | 0.058785 | 0.039435 |
|  | rs1954532 | C | T | 0.082625 | 0.017525 | 2.27E-06 | 0.052863 | 0.034074 | 0.120805 |
|  | rs28889936 | A | C | 0.114051 | 0.025284 | 3.37E-06 | -0.089233 | 0.045911 | 0.051946 |
|  | rs62422654 | C | T | 0.089846 | 0.019809 | 6.47E-06 | -0.032874 | 0.033327 | 0.323926 |
|  | rs72866977 | C | A | 0.130569 | 0.028167 | 5.63E-06 | -0.063326 | 0.050754 | 0.212143 |
|  | rs751183 | C | T | 0.077432 | 0.017222 | 6.85E-06 | 0.018889 | 0.035146 | 0.590965 |
|  | rs8076323 | A | G | 0.071539 | 0.015654 | 5.61E-06 | 0.022732 | 0.028933 | 0.432064 |
| *Ruminococcaceae_UCG-002* | rs10916131 | T | C | 0.069333 | 0.014675 | 2.87E-06 | 0.04874 | 0.036806 | 0.185423 |
|  | rs10927423 | A | C | 0.071362 | 0.014771 | 8.50E-07 | 0.024007 | 0.03553 | 0.499243 |
|  | rs10964441 | A | G | 0.14906 | 0.034486 | 7.45E-06 | -0.08355 | 0.044741 | 0.061843 |
|  | rs113147300 | G | A | 0.075842 | 0.016456 | 7.69E-06 | 0.014506 | 0.039581 | 0.714013 |
|  | rs11607472 | G | A | 0.078023 | 0.017632 | 7.19E-06 | 0.047731 | 0.054208 | 0.378587 |
|  | rs116974815 | A | C | 0.189731 | 0.039657 | 2.03E-06 | 0.000639 | 0.053267 | 0.990426 |
|  | rs11750293 | T | G | 0.05783 | 0.012051 | 1.76E-06 | -0.022838 | 0.028206 | 0.418129 |
|  | rs12463378 | G | A | 0.052206 | 0.011209 | 2.96E-06 | -0.017397 | 0.029583 | 0.556488 |
|  | rs15256 | C | T | 0.073238 | 0.016834 | 9.46E-06 | -0.026386 | 0.042193 | 0.531731 |
|  | rs55793120 | T | C | 0.137396 | 0.027414 | 4.81E-07 | -0.02485 | 0.057521 | 0.665733 |
|  | rs56030423 | A | G | 0.098287 | 0.021634 | 6.30E-06 | -0.02217 | 0.053151 | 0.676592 |
|  | rs57079348 | G | T | 0.076573 | 0.017282 | 7.22E-06 | -0.040897 | 0.058496 | 0.484457 |
|  | rs6542556 | A | G | 0.050974 | 0.011406 | 7.86E-06 | 0.01297 | 0.02795 | 0.642624 |
|  | rs6793778 | T | C | 0.055871 | 0.012526 | 9.81E-06 | -0.105558 | 0.030288 | 0.000492 |
|  | rs7120052 | A | C | 0.06248 | 0.013552 | 1.97E-06 | 0.002804 | 0.034694 | 0.935582 |
|  | rs7155595 | C | A | 0.056993 | 0.011699 | 1.15E-06 | -0.007783 | 0.029558 | 0.792324 |
|  | rs7249614 | G | A | 0.049278 | 0.011081 | 9.07E-06 | -0.000112 | 0.027985 | 0.996794 |
|  | rs7342369 | A | C | 0.05278 | 0.011602 | 5.66E-06 | -0.015036 | 0.031152 | 0.629334 |
|  | rs76847269 | A | G | 0.163508 | 0.035615 | 5.17E-06 | -0.179752 | 0.086503 | 0.037709 |
|  | rs77564310 | C | A | 0.071309 | 0.014086 | 3.29E-07 | -0.008687 | 0.03355 | 0.795698 |
|  | rs79016051 | T | C | 0.088775 | 0.018942 | 2.34E-06 | -0.044218 | 0.040166 | 0.270956 |
|  | rs882348 | G | A | 0.079995 | 0.017862 | 5.45E-06 | -0.032415 | 0.042164 | 0.442024 |
| MR, Mendelian randomization; PsA, Psoriatic arthritis; SNP, single nucleotide polymorphism; SE, standard error. | | | | | | | | | |

**Supplemental Table S3:** Directional horizontal pleiotropy assessed by intercept term in MR Egger regression of the association between gut microbiome and PsA.

| **Table S3 Directional horizontal pleiotropy assessed by intercept term in MR Egger regression of the association between gut microbiome and PsA.** | | | |
| --- | --- | --- | --- |
| **Bacterial taxa (exposure)** | **Egger_intercept** | **SE** | ***P*-value** |
| *Blautia* | 0.000 | 0.029 | 0.993 |
| *Butyricicoccus* | -0.072 | 0.028 | 0.042 |
| *Christensenellaceae_R-7_group* | -0.015 | 0.055 | 0.798 |
| *Defluviitaleaceae_UCG-011* | 0.073 | 0.051 | 0.196 |
| *Eubacterium_fissicatena_group* | 0.089 | 0.060 | 0.180 |
| *Family_XIII_AD3011_group* | 0.068 | 0.065 | 0.319 |
| *Fusicatenibacter* | 0.020 | 0.032 | 0.540 |
| *Methanobrevibacter* | -0.050 | 0.058 | 0.438 |
| *Oscillospira* | 0.165 | 0.058 | 0.029 |
| *Ruminococcaceae_UCG-002* | 0.007 | 0.022 | 0.758 |
| MR, Mendelian randomization; PsA, Psoriatic arthritis; SE, standard error. | | | |

**Supplemental Table S4:** MR-PRESSO analysis for the association between gut microbiome and PsA.

| **Table S4 MR-PRESSO analysis for the association between gut microbiome and PsA.** | | | | | | | | |
| --- | --- | --- | --- | --- | --- | --- | --- | --- |
| **Bacterial taxa (exposure)** | **MR Analysis** | **Causal Estimate** | **SD** | **T** | **P-value** | **RSSobs** | **Global test P-value** | **Remove SNP** |
| *Blautia* | MR-PRESSO | 0.309 | 0.116 | 2.671 | 0.020 | 8.133 | 0.876 | - |
| *Butyricicoccus* | MR-PRESSO | -0.390 | 0.218 | -1.791 | 0.116 | 16.169 | 0.142 | - |
| *Christensenellaceae_R-7_group* | MR-PRESSO | -0.162 | 0.230 | -0.704 | 0.499 | 21.456 | 0.062 | - |
| *Defluviitaleaceae_UCG-011* | MR-PRESSO | 0.272 | 0.151 | 1.798 | 0.110 | 14.919 | 0.204 | - |
| *Eubacterium_fissicatena_group* | MR-PRESSO | 0.247 | 0.089 | 2.787 | 0.024 | 10.065 | 0.495 | - |
| *Family_XIII_AD3011_group* | MR-PRESSO | 0.274 | 0.170 | 1.611 | 0.133 | 22.722 | 0.099 | - |
| *Fusicatenibacter* | MR-PRESSO | 0.227 | 0.098 | 2.319 | 0.033 | 11.111 | 0.906 | - |
| *Methanobrevibacter* | MR-PRESSO | 0.270 | 0.062 | 4.326 | 0.008 | 2.406 | 0.899 | - |
| *Oscillospira* | MR-PRESSO | -0.223 | 0.189 | -1.183 | 0.275 | 15.781 | 0.112 | - |
| *Ruminococcaceae_UCG-002* | MR-PRESSO | -0.233 | 0.107 | -2.182 | 0.041 | 24.743 | 0.411 | - |
| MR, Mendelian randomization; PsA, Psoriatic arthritis; SD, standard deviation; RSS_obs,_ observed residual sum of squares. | | | | | | | | |

**Supplemental Table S5:** The heterogeneity of gut microbiome IVs.

| **Table S5 The heterogeneity of gut microbiome IVs.** | | | | | | |  |  |  |
| --- | --- | --- | --- | --- | --- | --- | --- | --- | --- |
| **Bacterial taxa (exposure)** | | **Cochran's Q** | | **df** | ***P*-value** | |  |  |  |
| *Blautia* | | 6.794 | | 12 | 0.871 | |  |  |  |
| *Butyricicoccus* | | 12.070 | | 7 | 0.098 | |  |  |  |
| *Christensenellaceae_R-7_group* | | 16.649 | | 9 | 0.054 | |  |  |  |
| *Defluviitaleaceae_UCG-011* | | 10.955 | | 8 | 0.204 | |  |  |  |
| *Eubacterium_fissicatena_group* | | 7.900 | | 8 | 0.443 | |  |  |  |
| *Family_XIII_AD3011_group* | | 19.070 | | 12 | 0.087 | |  |  |  |
| *Fusicatenibacter* | | 9.857 | | 17 | 0.910 | |  |  |  |
| *Methanobrevibacter* | | 1.652 | | 5 | 0.895 | |  |  |  |
| *Oscillospira* | | 12.258 | | 7 | 0.092 | |  |  |  |
| *Ruminococcaceae_UCG-002* | | 22.587 | | 21 | 0.366 | |  |  |  |
| Df, degree of freedom. | | | | | | |  |  |  |
| Table S6 Full result of MR estimates for the association between PsA and gut microbiome. | | | | | | |  |  |  |
| **Bacterial taxa (outcome)** | **MR method** | | **No. of SNP** | | | **Beta** | | **SE** | ***P*-value** |
| *Blautia* | IVW | | 11 | | | 0.00 | | 0.02 | 0.85 |
|  | MR Egger | | 11 | | | -0.02 | | 0.03 | 0.59 |
|  | Weighted median | | 11 | | | 0.00 | | 0.02 | 0.94 |
|  | Weighted mode | | 11 | | | 0.00 | | 0.02 | 0.97 |
|  | Maximum likelihood | | 11 | | | 0.00 | | 0.01 | 0.83 |
|  | cML-MA-BIC | | 11 | | | 0.00 | | 0.01 | 0.82 |
| *Butyricicoccus* | IVW | | 11 | | | -0.01 | | 0.02 | 0.72 |
|  | MR Egger | | 11 | | | -0.07 | | 0.03 | 0.05 |
|  | Weighted median | | 11 | | | -0.03 | | 0.02 | 0.07 |
|  | Weighted mode | | 11 | | | -0.03 | | 0.02 | 0.11 |
|  | Maximum likelihood | | 11 | | | -0.01 | | 0.01 | 0.67 |
|  | cML-MA-BIC | | 11 | | | 0.00 | | 0.02 | 0.79 |
| *Christensenellaceae_R-7_group* | IVW | | 11 | | | 0.00 | | 0.02 | 0.93 |
|  | MR Egger | | 11 | | | -0.02 | | 0.04 | 0.69 |
|  | Weighted median | | 11 | | | -0.02 | | 0.02 | 0.42 |
|  | Weighted mode | | 11 | | | -0.03 | | 0.02 | 0.23 |
|  | Maximum likelihood | | 11 | | | 0.00 | | 0.01 | 0.91 |
|  | cML-MA-BIC | | 11 | | | -0.01 | | 0.02 | 0.56 |
| *Defluviitaleaceae_UCG-011* | IVW | | 11 | | | -0.04 | | 0.02 | 0.07 |
|  | MR Egger | | 11 | | | -0.02 | | 0.05 | 0.72 |
|  | Weighted median | | 11 | | | -0.04 | | 0.03 | 0.17 |
|  | Weighted mode | | 11 | | | -0.04 | | 0.03 | 0.21 |
|  | Maximum likelihood | | 11 | | | -0.04 | | 0.02 | 0.07 |
|  | cML-MA-BIC | | 11 | | | -0.04 | | 0.02 | 0.04 |
| *Eubacterium_fissicatena_group* | IVW | | 10 | | | 0.01 | | 0.04 | 0.74 |
|  | MR Egger | | 10 | | | 0.03 | | 0.09 | 0.71 |
|  | Weighted median | | 10 | | | 0.01 | | 0.04 | 0.83 |
|  | Weighted mode | | 10 | | | 0.01 | | 0.04 | 0.72 |
|  | Maximum likelihood | | 10 | | | 0.01 | | 0.03 | 0.66 |
|  | cML-MA-BIC | | 10 | | | 0.01 | | 0.03 | 0.65 |
| *Family_XIII_AD3011_group* | IVW | | 11 | | | 0.02 | | 0.02 | 0.16 |
|  | MR Egger | | 11 | | | 0.01 | | 0.03 | 0.66 |
|  | Weighted median | | 11 | | | 0.02 | | 0.02 | 0.44 |
|  | Weighted mode | | 11 | | | 0.01 | | 0.02 | 0.50 |
|  | Maximum likelihood | | 11 | | | 0.02 | | 0.02 | 0.16 |
|  | cML-MA-BIC | | 11 | | | 0.02 | | 0.02 | 0.20 |
| *Fusicatenibacter* | IVW | | 11 | | | 0.02 | | 0.01 | 0.24 |
|  | MR Egger | | 11 | | | 0.01 | | 0.03 | 0.70 |
|  | Weighted median | | 11 | | | 0.01 | | 0.02 | 0.42 |
|  | Weighted mode | | 11 | | | 0.01 | | 0.02 | 0.42 |
|  | Maximum likelihood | | 11 | | | 0.02 | | 0.01 | 0.24 |
|  | cML-MA-BIC | | 11 | | | 0.01 | | 0.01 | 0.40 |
| *Methanobrevibacter* | IVW | | 10 | | | 0.00 | | 0.03 | 0.93 |
|  | MR Egger | | 10 | | | 0.04 | | 0.07 | 0.61 |
|  | Weighted median | | 10 | | | 0.00 | | 0.04 | 0.92 |
|  | Weighted mode | | 10 | | | 0.01 | | 0.04 | 0.83 |
|  | Maximum likelihood | | 10 | | | 0.00 | | 0.03 | 0.93 |
|  | cML-MA-BIC | | 10 | | | 0.01 | | 0.03 | 0.87 |
| *Oscillospira* | IVW | | 11 | | | 0.03 | | 0.02 | 0.09 |
|  | MR Egger | | 11 | | | 0.02 | | 0.04 | 0.72 |
|  | Weighted median | | 11 | | | 0.02 | | 0.02 | 0.51 |
|  | Weighted mode | | 11 | | | 0.01 | | 0.03 | 0.61 |
|  | Maximum likelihood | | 11 | | | 0.03 | | 0.02 | 0.07 |
|  | cML-MA-BIC | | 11 | | | 0.03 | | 0.02 | 0.13 |
| *Ruminococcaceae_UCG-002* | IVW | | 11 | | | 0.01 | | 0.02 | 0.44 |
|  | MR Egger | | 11 | | | -0.02 | | 0.04 | 0.70 |
|  | Weighted median | | 11 | | | -0.01 | | 0.02 | 0.53 |
|  | Weighted mode | | 11 | | | -0.01 | | 0.02 | 0.45 |
|  | Maximum likelihood | | 11 | | | 0.01 | | 0.01 | 0.34 |
|  | cML-MA-BIC | | 11 | | | 0.01 | | 0.02 | 0.52 |
| MR, Mendelian randomization; PsA, Psoriatic arthritis; SNP, single nucleotide polymorphism; IVW, inverse variance weighted. | | | | | | | | | |
