# Supplementary material for: Genetic causal relationship between gut microbiome and psoriatic arthritis: a bidirectional two-sample Mendelian randomization study
Source: Front Microbiol. 2023 Oct 31;14:1265786. doi: 10.3389/fmicb.2023.1265786 (PMC10644104; doi:10.3389/fmicb.2023.1265786)

**Legends for supplementary materials:**

**Supplemental Figure S1:** Forest plot (A), sensitivity analysis (B), scatter plot (C), and funnel plot (D) of the causal effect of *Defluviitaleaceae_UCG-011*on PsA risk.


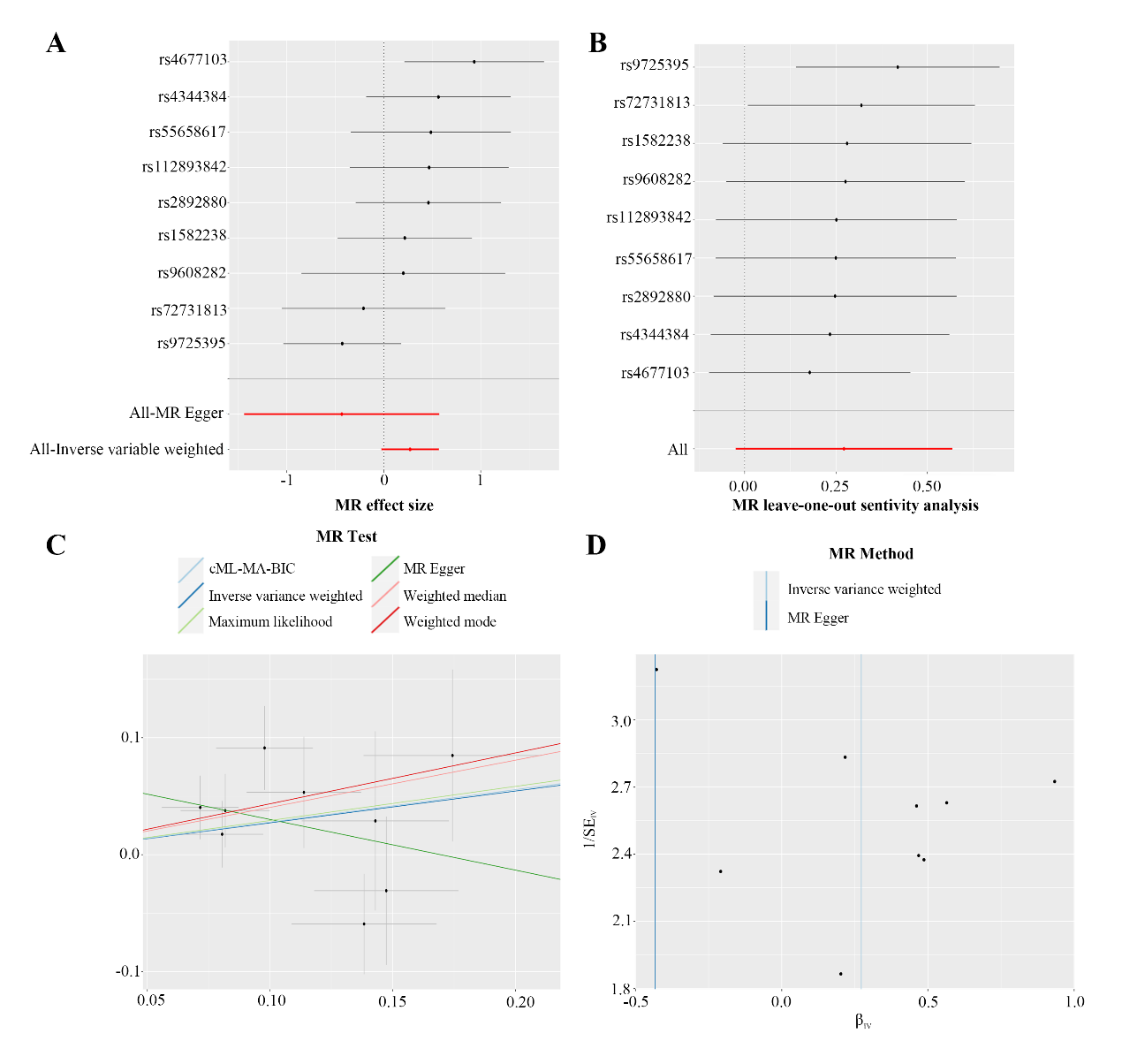


**Supplemental Figure S2:** Forest plot (A), sensitivity analysis (B), scatter plot (C), and funnel plot (D) of the causal effect of *Eubacterium_fissicatena_group* on PsA risk.

**
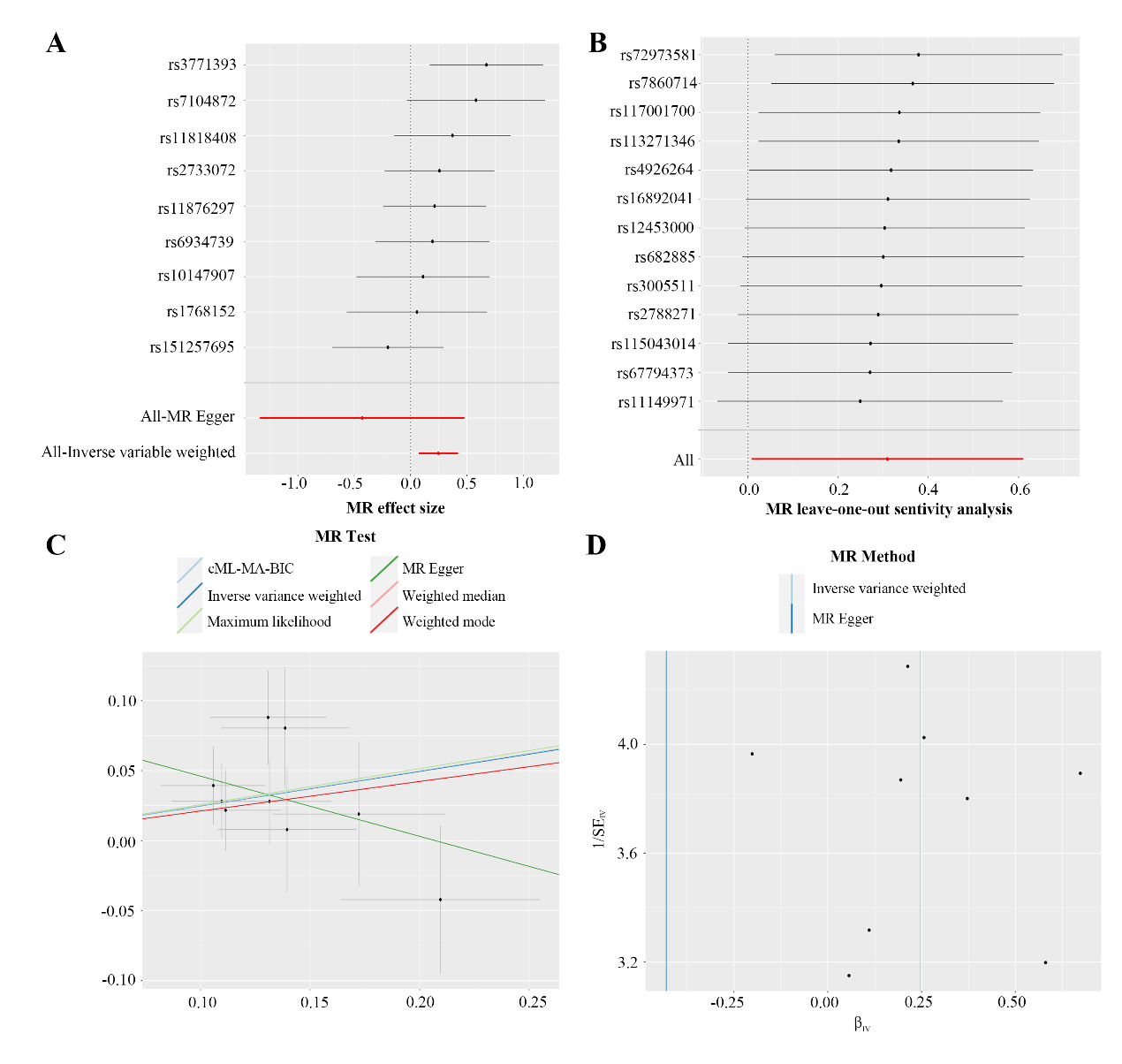
**

**Supplemental Figure S3:** Forest plot (A), sensitivity analysis (B), scatter plot (C), and funnel plot (D) of the causal effect of *Family_XIII_AD3011_group* on PsA risk.

**
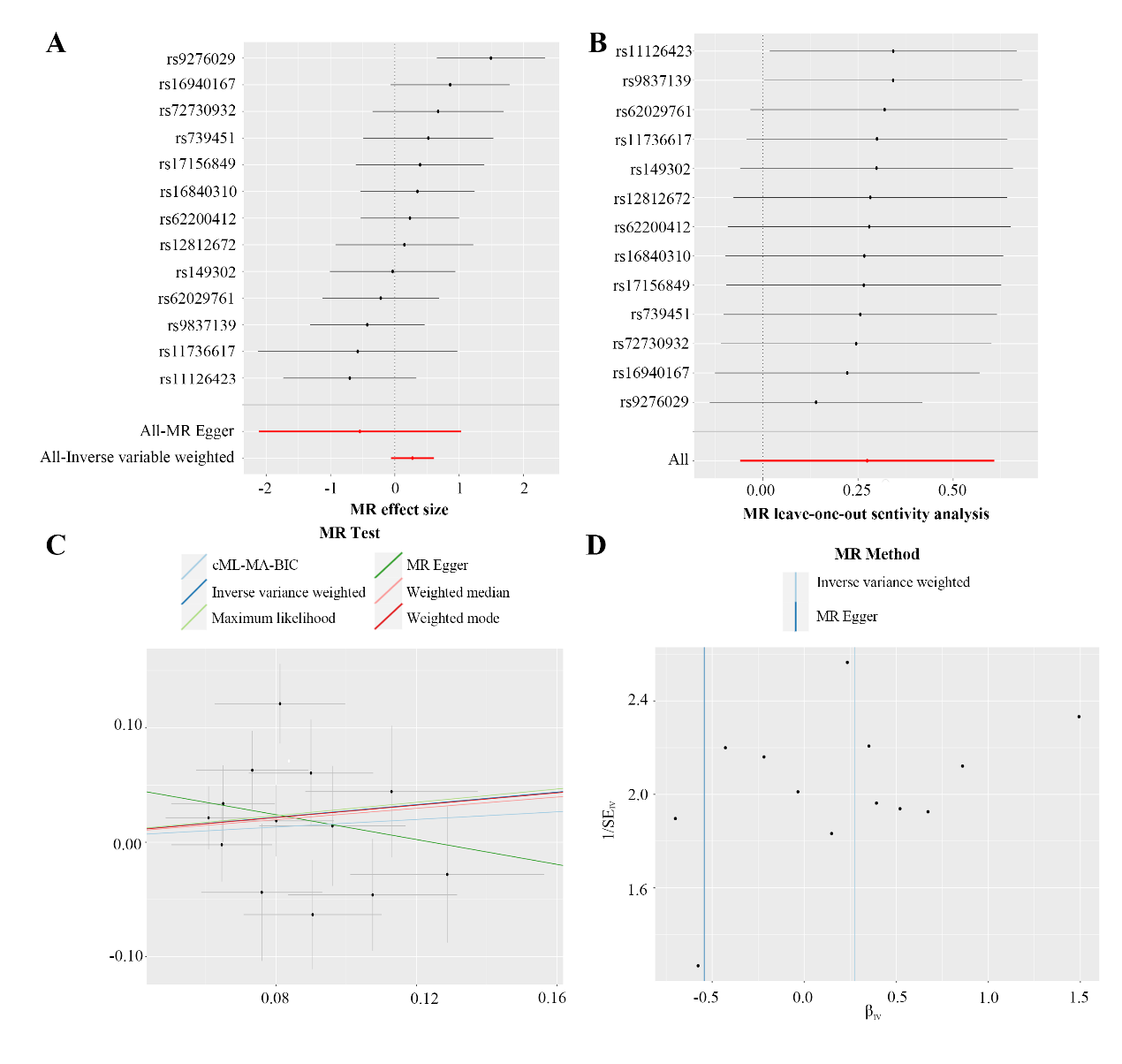
**

**Supplemental Figure S4:** Forest plot (A), sensitivity analysis (B), scatter plot (C), and funnel plot (D) of the causal effect of *Fusicatenibacter* on PsA risk.

**
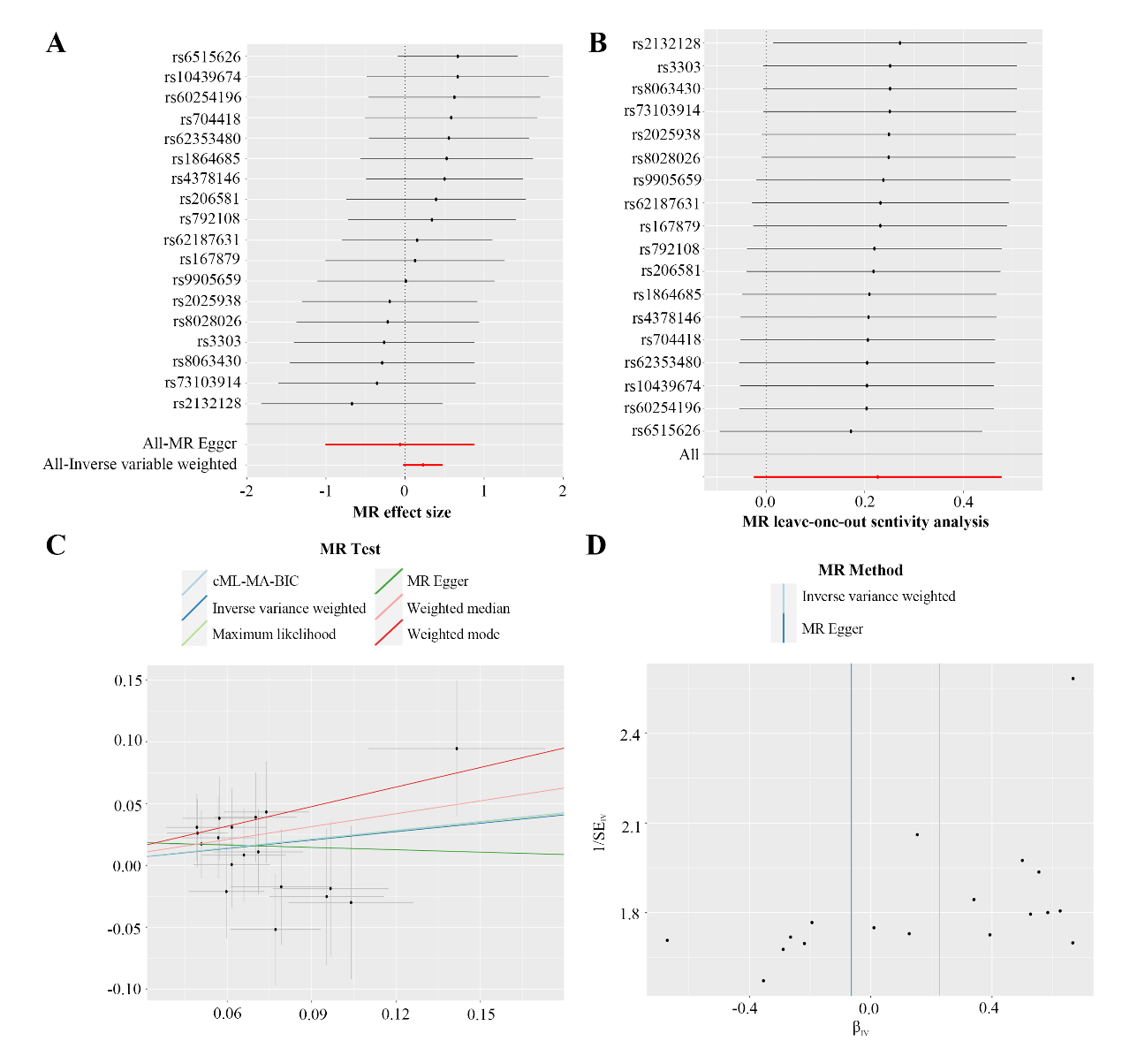
**

**Supplemental Figure S5:** Forest plot (A), sensitivity analysis (B), scatter plot (C), and funnel plot (D) of the causal effect of *Methanobrevibacter* on PsA risk.


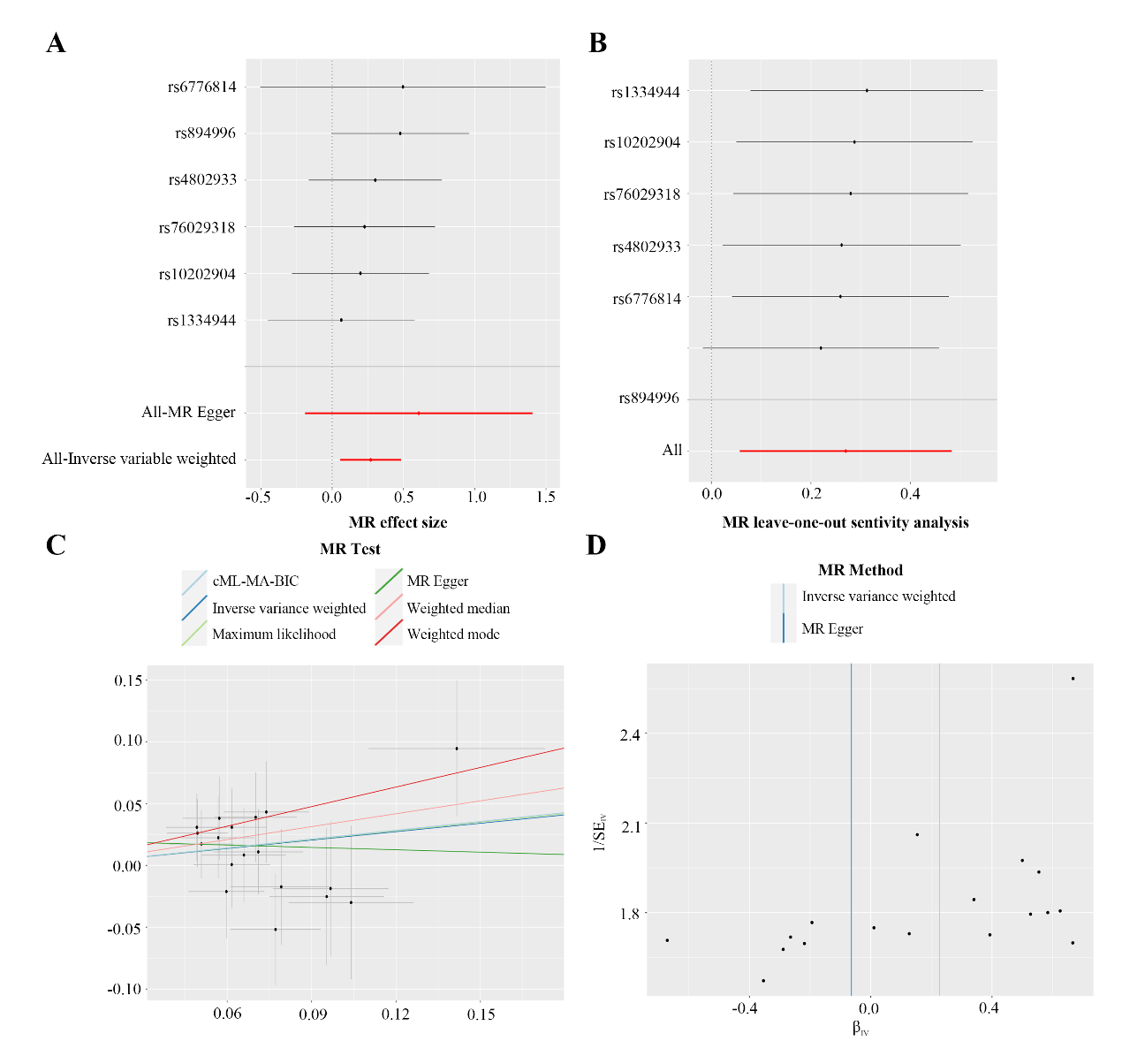


**Supplemental Figure S6:** Forest plot (A), sensitivity analysis (B), scatter plot (C), and funnel plot (D) of the causal effect of *Butyricicoccus* on PsA risk.

**
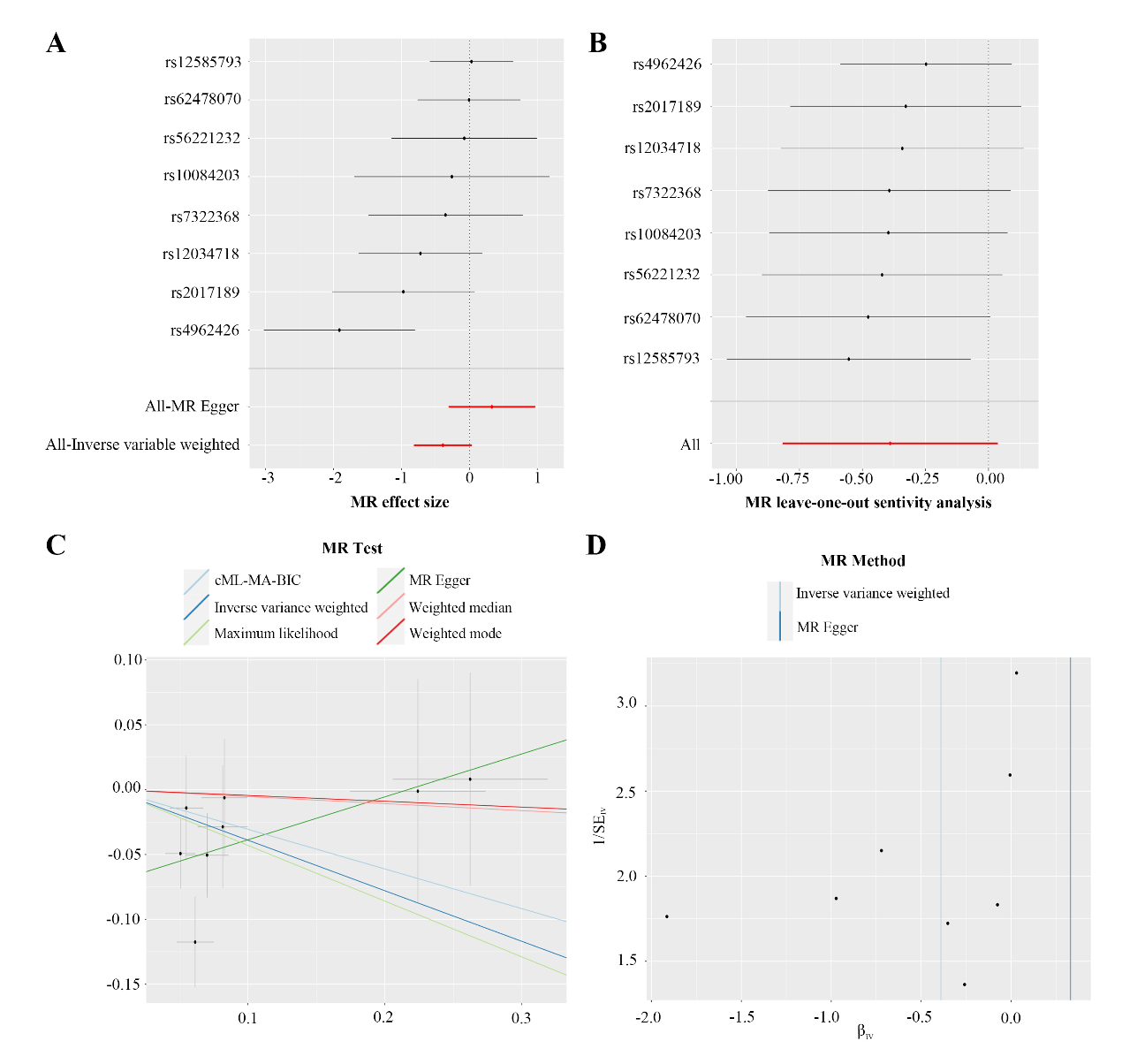
**

**Supplemental Figure S7:** Forest plot (A), sensitivity analysis (B), scatter plot (C), and funnel plot (D) of the causal effect of *Christensenellaceae_R-7_group* on PsA risk.

**
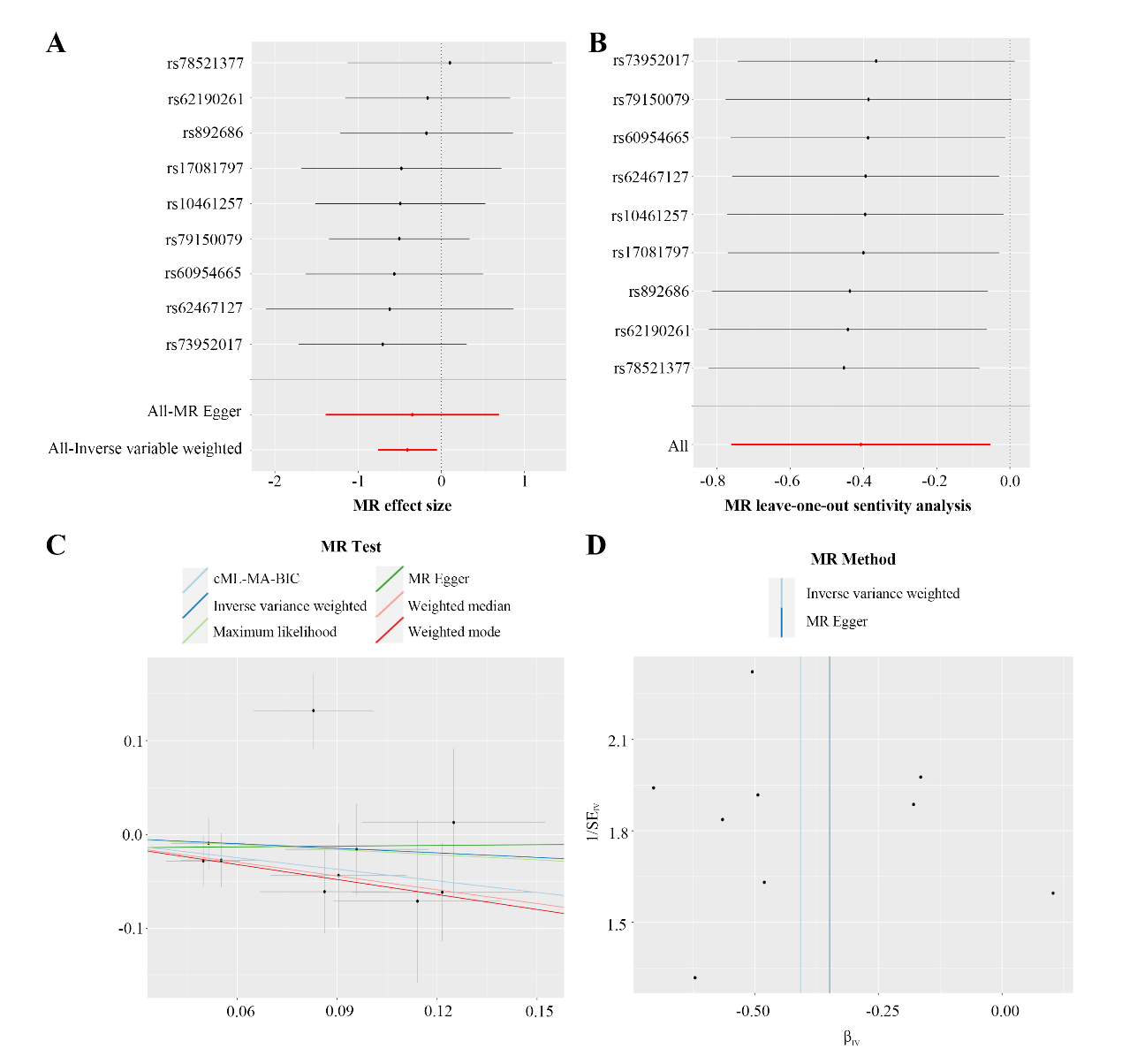
**

**Supplemental Figure S8:** Forest plot (A), sensitivity analysis (B), scatter plot (C), and funnel plot (D) of the causal effect of *Oscillospira* on PsA risk.

**
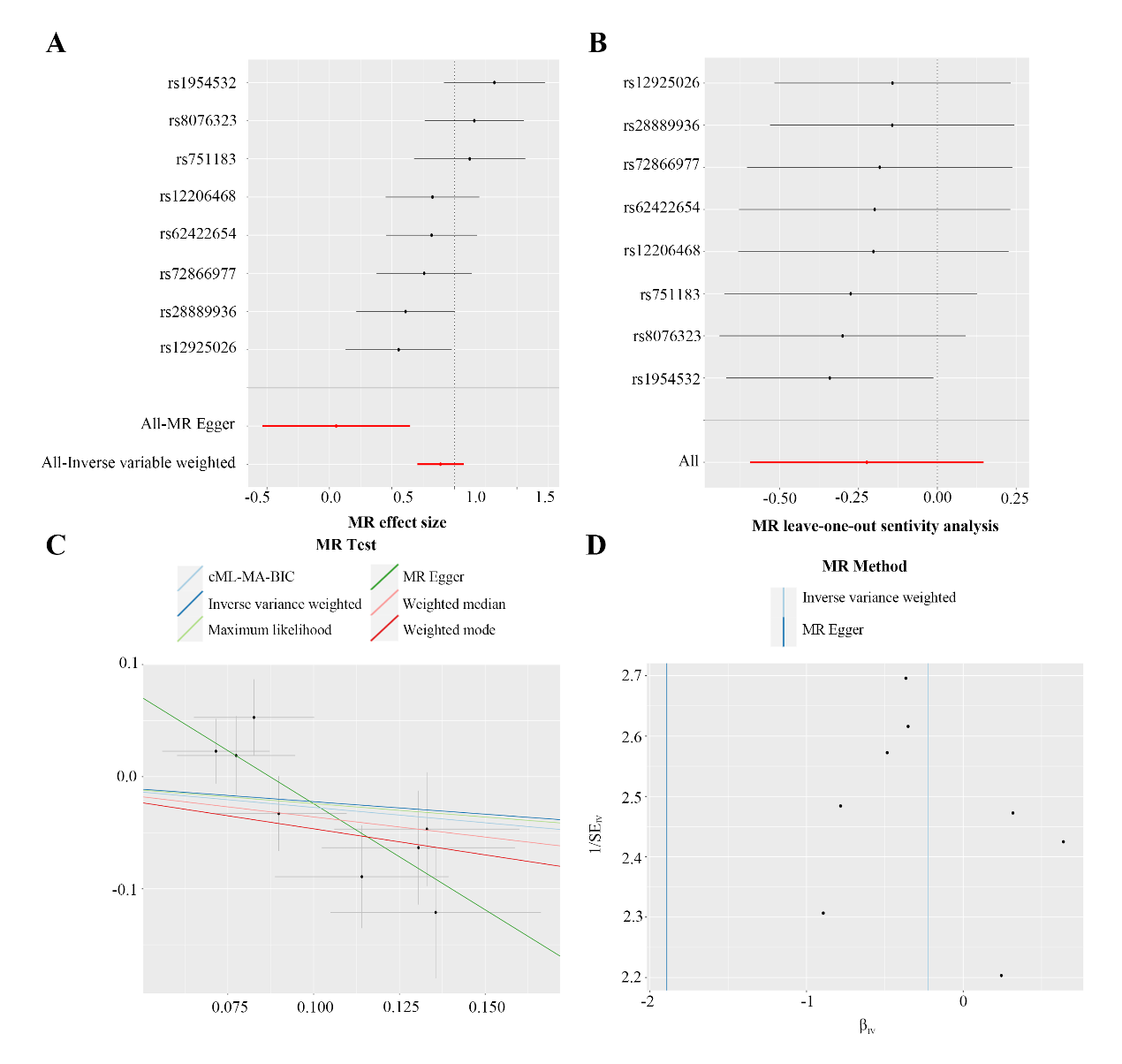
**

**Supplemental Figure S9:** Forest plot (A), sensitivity analysis (B), scatter plot (C), and funnel plot (D) of the causal effect of *Ruminococcaceae_UCG-002* on PsA risk.


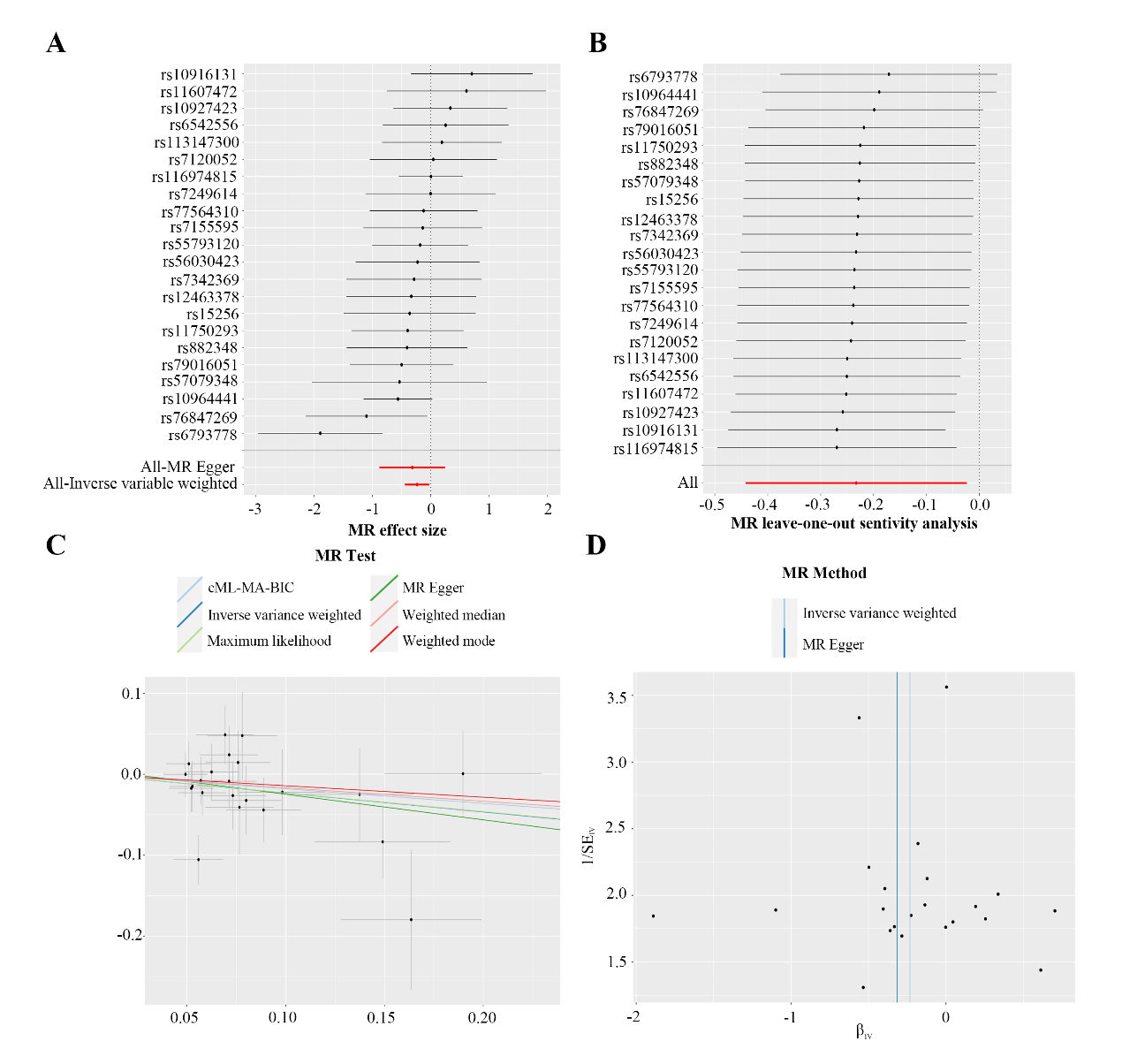

Supplement: Supplementary file 2 [file Data_Sheet_2.DOCX]
